# The role of the California tier system in controlling population mobility during the COVID-19 pandemic

**DOI:** 10.1186/s12889-023-15858-7

**Published:** 2023-05-18

**Authors:** Emilie Schwarz, Lara Schwarz, Anaïs Teyton, Katie Crist, Tarik Benmarhnia

**Affiliations:** 1 École des Hautes Études en Santé Publique, Paris, France; 2 School of Public Health, San Diego State University, La Jolla, San Diego, CA USA; 3 Herbert Wertheim School of Public Health and Human Longevity Science, University of California San Diego, La Jolla, San Diego, CA USA; 4 Scripps Institution of Oceanography, University of California San Diego, La Jolla, San Diego, CA USA

**Keywords:** COVID-19, Mobility, Policy evaluation, California, Mobile devices

## Abstract

**Background:**

Policies to restrict population mobility are a commonly used strategy to limit the transmission of contagious diseases. Among measures implemented during the COVID-19 pandemic were dynamic stay-at-home orders informed by real-time, regional-level data. California was the first state in the U.S. to implement this novel approach; however, the effectiveness of California’s four-tier system on population mobility has not been quantified.

**Methods:**

Utilizing data from mobile devices and county-level demographic data, we evaluated the impact of policy changes on population mobility and explored whether demographic characteristics explained variability in responsiveness to policy changes. For each California county, we calculated the proportion of people staying home and the average number of daily trips taken per 100 persons, across different trip distances and compared this to pre-COVID-19 levels.

**Results:**

We found that overall mobility decreased when counties moved to a more restrictive tier and increased when moving to a less restrictive tier, as the policy intended. When placed in a more restrictive tier, the greatest decrease in mobility was observed for shorter and medium-range trips, while there was an unexpected increase in the longer trips. The mobility response varied by geographic region, as well as county-level median income, gross domestic product, economic, social, and educational contexts, the prevalence of farms, and recent election results.

**Conclusions:**

This analysis provides evidence of the effectiveness of the tier-based system in decreasing overall population mobility to ultimately reduce COVID-19 transmission. Results demonstrate that socio-political demographic indicators drive important variability in such patterns across counties.

**Supplementary Information:**

The online version contains supplementary material available at 10.1186/s12889-023-15858-7.

## Introduction

Population mobility is a critical consideration when examining the spread of infectious diseases from a public health perspective. Mobility, or the distances that people cover on a daily basis and the frequency of their trips, is an indication of their potential exposure to infected individuals (for communicable diseases) [1]. When officials are attempting to contain an infectious agent like the SARS-CoV-2 virus in a population, non-pharmaceutical interventions like government orders that restrict human mobility are commonly put in place to minimize exposure and viral spread. In the context of COVID-19, local and federal governments around the world applied various measures to limit the transmission of the virus by reducing population mobility. The SARS-CoV-2 virus rapidly spread globally largely from airborne transmission and had a high health burden, where its clinical presentation ranged from mild to severe symptoms including pneumonia, organ damage, and death, making it important to set policies to reduce its spread [2, 3]. However, the unprecedented nature of this global pandemic made guidance challenging to establish, partly due to a lack of clear evidence of the effectiveness of various measures.

A range of policies were implemented at various levels of government, including the use of face masks, physical distancing, limiting the capacity of indoor spaces, closing non-essential businesses and spaces, improving indoor ventilation, cleaning and disinfecting surfaces, and monitoring individual health through temperature-taking and COVID-19 testing [4–7]. Along with encouraging these policies, many governments also implemented stay-at-home orders to minimize the spread of infection. To various degrees, these orders required residents to stay at home, with exceptions for necessary or critical tasks [8]. Compared to regions where stay-at-home orders were not implemented, areas with stay-at-home orders had a reduction in the number of COVID-19 cases and deaths as shown as early as five days after implementation, thus minimizing viral spread [9, 10]. While the main mechanism that may contribute to such a reduction in COVID-19 is likely driven by changes in mobility patterns, there is little evidence to demonstrate the effect of these measures on mobility.

Several previous studies have focused on the effects of mobility restriction policies on COVID-19 transmission rates and have largely identified that mobility restrictions led to reductions in COVID-19 transmission [7, 11–21]. Fewer studies, however, have assessed the impacts of population mobility from COVID-19 policies. We identified 22 papers that have investigated this relationship, which are provided in Table S1. Seven of these studies assessed the impact of these policies on mobility within the United States (U.S.) only [22–26], while fifteen have explored this relationship in other parts of the world including China, Canada, Poland, Italy, France, Hungary, Greece, and on a global scale [7, 16, 27–40]. Certain countries, including China, the United Kingdom, South Africa, and Israel, utilized regional-level, real-time evidence to implement various levels of mobility restrictions to reduce the spread of COVID-19 [41–43]. To assess the efficacy of these measures, different approaches have been used to estimate mobility. One study conducted in China utilized traffic congestion and subway ridership frequency as a proxy for human mobility, while others relied on anonymized cell phone data or Google mobility data [32]. Regardless of the approach used, all of the studies concluded that these policies reduced mobility and highlighted their overall effectiveness in decreasing COVID-19 transmission. California was the first state in the U.S. to implement a more nuanced policy, which differed from state-wide stay-at-home orders due to its county-level criteria for determining policy restrictions. Moreover, few countries globally utilized a tier system policy (e.g., Italy), and those that did utilized differing policies (e.g., a three-tiered system in Italy using three colors for intensifying restrictions: yellow, orange, and red), with most countries opting to use lockdown policies without a tier system, making the California tier system distinctive [29].

On August 30th, 2020, California introduced the Blueprint for a Safer Economy, a comprehensive, county-by-county tier-system to control virus spread through policy restrictions, based on county-specific COVID-19 test positivity and case rates [44]. This tier system was put forth by California Governor Gavin Newsom as an evidence-based approach to combat the COVID-19 pandemic, considering local indicators of viral transmission. The California system included four tiers, with tier one being the most restrictive (purple), tier two (red) being restrictive, tier three (orange) being less restrictive, and tier four (yellow) being the least (Supplementary table S2). As this system was color-coded, it allowed for a clear and accessible indicator of COVID-19 risks for people and businesses in California. The tier of each county was evaluated based on epidemiological evidence of COVID-19 risks. On October 6th, 2020, California implemented an additional health equity metric that required counties to make tangible efforts to eliminate disparities by improving COVID-19 positivity rates in the most disadvantaged communities before moving to a less restrictive tier [45]. Once California began administering vaccines, the tier system shifted to adjust for vaccines being administered as well as the absolute case number from April 20th, 2021, onward.

Restrictions for businesses in each county corresponded to the tier for which they were classified; for instance, at Tier 1, non-essential indoor businesses were closed, and essential indoor businesses were reduced to a maximum capacity of 25%, while at Tier 4, most indoor businesses were open with some modifications. To our knowledge, the impact of this tier system on population mobility has not been evaluated. Furthermore, the role of different socio-political and demographic indicators may provide insight as to why mobility patterns vary from county to county. In counties that have a higher proportion of essential workforce, such as farmworkers for example, people may not have the privilege to work from home and therefore the mobility levels may not change with lockdowns, while counties with fewer essential workers may have more choice in restricting their mobility. Previous studies have also identified that certain sociodemographic characteristics, may be linked to disproportional COVID-19 exposure risk and impact, and these differences may in part explain differences in the relationship between policy implementation and mobility changes within and between counties [46, 47]. Understanding spatial differences in the effectiveness of policies aimed to reduce population mobility and what drives these variations can be important to tailor actions based on county-level demographics and populations. Understanding the response of populations at the county level can be used to promote equitable policies that account for disparities in exposure risk and associated protective measures. Additionally, evaluating the effects of these measures on shorter and longer distance travel can be helpful to further understand how populations change their travel behaviors and unintended impacts that may result from these policies. Addressing these knowledge gaps could provide invaluable insights to improve implementation of future population-wide policies and enhance public health emergency preparedness, management, and response.

This paper aims to evaluate the effect that the Blueprint for a Safer Economy, referred to subsequently as the California tier system, had on population mobility, including the daily proportion of the population not staying at home and the average number of trips by distance during its implementation from August 31st, 2020 to June 15th, 2021 in the peak of the COVID-19 pandemic. Secondly, this study aims to understand if demographic characteristics at the county level explain differences in how California counties’ mobility patterns changed in response to changing restrictions during policy implementation.

## Methods

### Data sources

Daily travel data including the population not staying at home and trips by distance travelled at the county level was downloaded from the U.S. Bureau of Transportation Statistics [48]. This travel information for each county in the U.S. was estimated by the Maryland Transportation Institute and Center for Advanced Transportation Technology Laboratory at the University of Maryland using an anonymized panel of mobile device data that was aggregated from several sources [48]. This includes data from mobile devices across the U.S. that meet data quality standards including temporal frequency and spatial accuracy of anonymized location point observations, temporal coverage and representativeness for the device, and spatial representativeness of the sample. A multi-level weighting method expanded the study sample to county-level population estimates and the results are estimated to have the best representativeness. The data extracted for this study included daily estimates of the population staying at home, the population not staying at home, and, when they leave home, the average number of trips by distance in miles (< 1, 1–3, 3–5, 5–10, 10–25, 25–50, 50–100, 100–250, 250–500, and 500+). Any movement involving a stay of longer than 10 min at an anonymized location away from home (which was imputed at the weekly level) was considered a trip; multiple stops of longer than 10 min before returning home were estimated as multiple trips. All modes of transportation are captured as trips including driving, rail, and air travel. This dataset began in January 2019 and data used for this study went from August 31st, 2020 through June 15th, 2021, when the tier system stopped, and California was fully reopened. Data from 2019 were additionally utilized for this analysis. Data on the California tier system was downloaded from the California Department of Public Health (CDPH) [45]. Each county in California was classified into a tier [1–4] based on indicators of COVID-19 transmission and risk using test positivity and adjusted case rates every week from August 31st, 2020, to June 15th, 2021. All metrics were based on county population size, and adjustments were made for small counties (defined as having less than 106,000 residents), including an exemption from adjusted rates based on testing volume and meeting health equity metrics that specify that test positivity rates in disadvantaged neighborhoods are not different from overall county estimates. Starting April 20th, 2021, absolute case number and vaccine coverage were also considered for moving counties to a less restrictive tier if the prior assessment prevented it from changing tiers. The CDPH assessed case rates and test positivity of the county every Monday and shared classification with counties every Tuesday, which would go into effect the next day (Wednesday). If it was determined that a county needed to move to a more restrictive tier, the county had three days, starting on Wednesday, to implement any changes unless immediate action was merited based on extreme circumstances. A county had to remain in a tier for a minimum of three weeks to advance to a less restrictive tier and could only move forward one tier at a time (even if metrics would indicate skipping tiers).

When a county was considered to have widespread COVID-19 transmission, it was categorized in the most restrictive tier (Purple, tier 1), and key restrictions included keeping social gatherings to a maximum of three households, not allowing indoor seated events, restricting gyms and restaurants to outdoors, and closing bars. In the Red tier (tier 2), when there was considered to be substantial transmission, a maximum of 25 people were allowed to gather outdoors, and indoor gatherings were allowed with modifications, although strongly discouraged. In this tier, gyms were allowed to open at 10% capacity and restaurants at 25% capacity, and bars remained closed. In the 3rd tier (Orange), 50 people were allowed to gather outdoors, gyms opened at 25% capacity and restaurants at 50%, while offices started to open indoors with some modification (although remote work continued to be strongly encouraged); bars also were allowed to open for outdoor use. The least restrictive tier (Yellow, tier 4) allowed outdoor gatherings of up to 100 people, gyms and restaurants open at 50% capacity, and bars to open indoors at 25% capacity. More details about what was allowed or restricted in each tier criteria are provided in the supplementary materials (Table S2).

### Analytical strategy

To understand the change in mobility resulting from the California Tier system, we first computed a population-standardized measure for each mobility variable by dividing the population staying at home and the number of trips by the total population (sum of population staying at home and population not staying at home) and multiplying by 100. These population-standardized measures represent the proportion of people staying home and the average number of daily trips taken for every 100 persons for each distance category. These estimates were averaged at the weekly level for each county, for a total of 83 weeks assessed for each of the 58 California counties, and a difference for every week was computed using 2019 estimates as a baseline to estimate a robust difference as done in previous papers [49, 50]. Such robust differences target the mobility pattern that would have been observed in the absence of the California Tier system restrictions for each week in 2020 (using weekly estimates from the previous year). A difference in average mobility at the weekly level was used as the outcome of interest in our models. It should be noted that the 1st of January was considered the first day of the first week of each year, and weekly measures were computed accordingly. With this, weeks started on a different weekday each year (Tuesday in 2019, Wednesday in 2020, and Friday in 2021). The week following the tier system evaluation was used for analysis; for example, the first week where the tier system was evaluated on the 30th of August 2020 corresponded to mobility data from September 2nd to 8th, 2020. A sensitivity analysis was conducted considering the first Wednesday and Friday as the first day of the week, given that the tier system went into effect on Wednesdays, and counties had three days to implement changes when moving into a more restrictive tier.

The county classification every week was collected, and any change to a higher tier level was considered to be less restrictive (i.e., dichotomous variable was utilized, where any change to a higher tier level was recoded as 1, no change or any change to a lower tier level was recoded as a 0), while any change to a lower tier level was considered more restrictive (i.e., any change to a lower tier level = 1, no change or any change to a higher tier level = 0). A linear model with fixed effects was applied with the change in tier level as the independent variable and the population-adjusted difference mobility measure as the dependent variable, with fixed effects (for the intercept and the slope) at the county level to compare weekly changes only within each county. The regression coefficients considering the effect of tier system changes on the population not staying at home for each county were extracted for further analysis.

### Demographic comparisons

A meta-regression was applied to understand the association between county-level demographics and variation in mobility in response to tier system implementation. County-level demographic data was downloaded from the California State Association of Counties [51], 2019 Census estimates, the California Healthy Places Index [52], and the New York Times recall election results [53]. County-level coefficients indicating the effect of a more restrictive tier were regressed with each demographic variable of interest and plotted to explore which characteristics explain the differences in effect observed between counties in California. Variables considered to be potentially related to a tier system response were the gross domestic product (GDP, as a measure of economic activity), median income, economic context (a measure of employment rates, per capita income, and poverty levels), social context (a measure of voter participation and census response), educational context (a measure of preschool enrollment, bachelor’s education, and high school enrollment), the number of farms in each county (an indicator for the agricultural workforce) and the proportion of each county that voted “Yes” to recall Governor Gavin Newsom. Many California residents petitioned for a California Gubernatorial Recall Election as a result of the tier system implemented by Governor Gavin Newsom because of its impacts on the California economy, which required many businesses to shut down or operate at a limited capacity. Census level indicators from the Healthy Places Index were aggregated at the county level by the calculation of population-weighted means. Data compilation and linear regressions were conducted in STATA 16 SE, while the meta-regression was conducted with R 4.1.0.

## Results

### Descriptive statistics

Table 1 provides the descriptive statistics regarding demographics, mobility, and recall vote information averaged for all counties in California. The average GDP is $51.7 billion, and the average median income is $77,470 for all counties. On average, there are 4.17 farms per 1,000 people. Over the course of the tier system implementation, an average of 72.87 individuals per 100 people were not staying at home, and the average number of trips that individuals took during the tier system implementation from August 31st, 2020, to June 15th, 2021, was 261.51 per 100 persons. These trips ranged from less than 1 mile which occurred, on average, at a frequency of 64.77 trips per 100 persons, to over 500 miles, which occurred less frequently with an average of 0.26 trips per 100 persons. Most trips (95.4%) remained under 50 miles, and a predominant number of trips were 3 miles or shorter. Across counties, an average of 47.14% of persons voted yes for the Gavin Newsom recall. County-specific demographics are presented in the supplementary material (Table S3).

**Table 1:** Average characteristics of California counties including mobility and county level socio-demographics

Mobility	Mean (standard deviation)	Demographics	Mean (standard deviation)
Population not staying at home (per 100)	72.87 (5.47)	GDP (billions)	51.7 (119)
Number of trips (per 100 persons)	261.51 (51.28)	Median income	77,470 (22,622)
<1 mile	64.77 (23.04)	# farms/1000 persons	4.17 (8.75)
1–3 miles	64.45 (14.27)	Recall vote	47.14 (16.30)
3–5 miles	29.20 (7.64)	Economic	-0.25 (0.52)
5–10 miles	33.61 (9.72)	Education	-0.23 (0.68)
10–25 miles	38.77 (13.20)	Social	0.22 (0.40)
25–50 miles	18.75 (8.44)	Age 65+	16.4 (3.98)
50–100 miles	8.13 (4.83)		
100–250 miles	3.03 (1.84)		
250–500 miles	0.54 (0.38)		
> 500 miles	0.26 (0.43)		

**Fig. 1:**
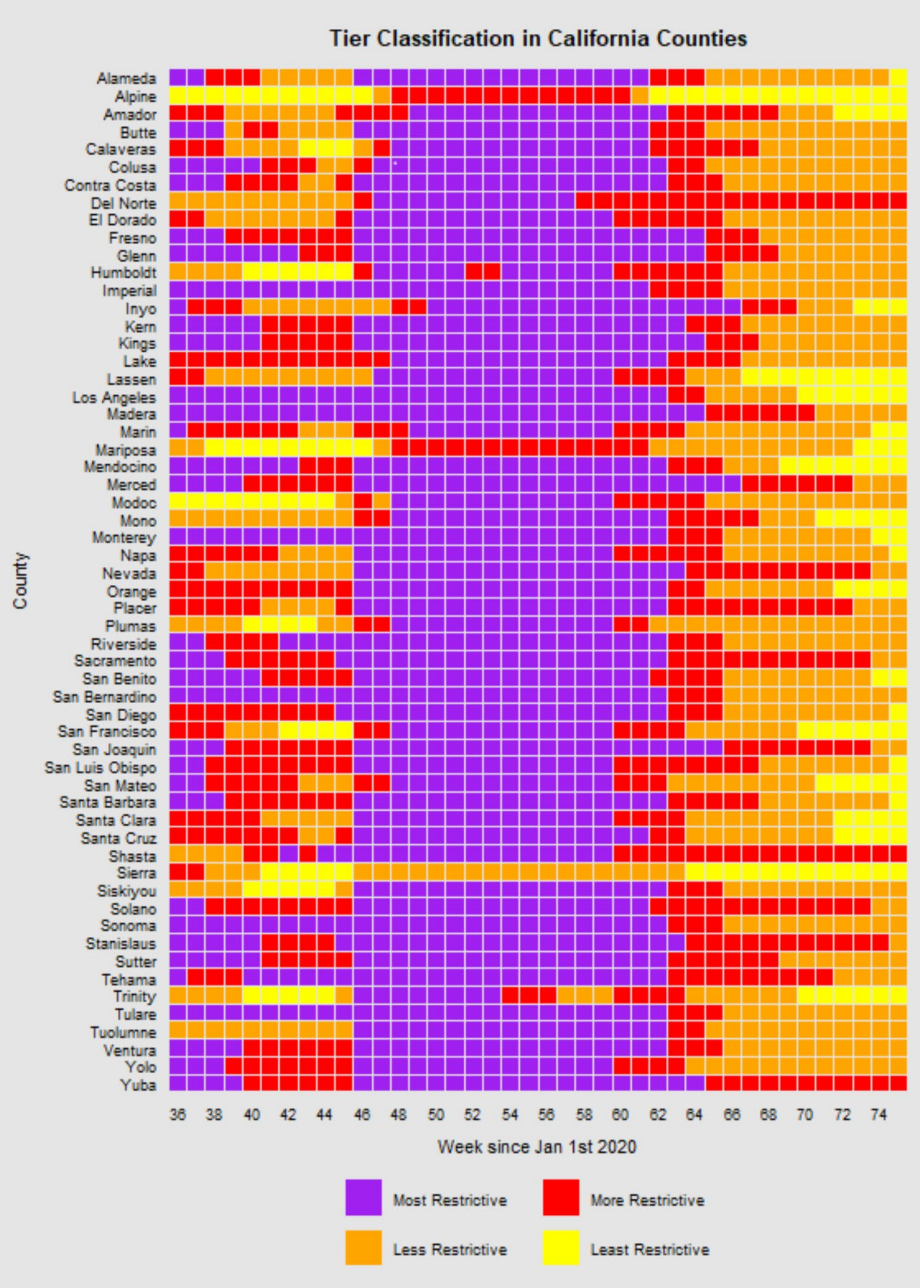
California tier changes by county from August 31st, 2020, to June 15th 2021 during implementation of the Blueprint for a Safer Economy (tier system)

Figure 1 depicts the changes in tier restrictions across the duration of the California tier system implementation for each county. The majority of counties (81%) began in the more restrictive tiers (red or purple) at the beginning of the tier system implementation, which tended to relax across the ensuing 3–6 weeks. Most counties, except Alpine, Mariposa, and Sierra counties, were in the purple (most restrictive) tier from November 2020 until late March 2021. This coincides with a large portion of the population qualifying to receive the vaccine, and most counties except for Del Norte, Shasta, and Yuba counties were in the less restrictive tiers (yellow and orange) by the time the tier system was retired on June 15, 2021. Most measures of mobility decreased on average in 2020 compared to 2019 and increased again in the first months (January- July) of 2021 (Table S4).

### Geographic differences in mobility

**Fig. 2:**
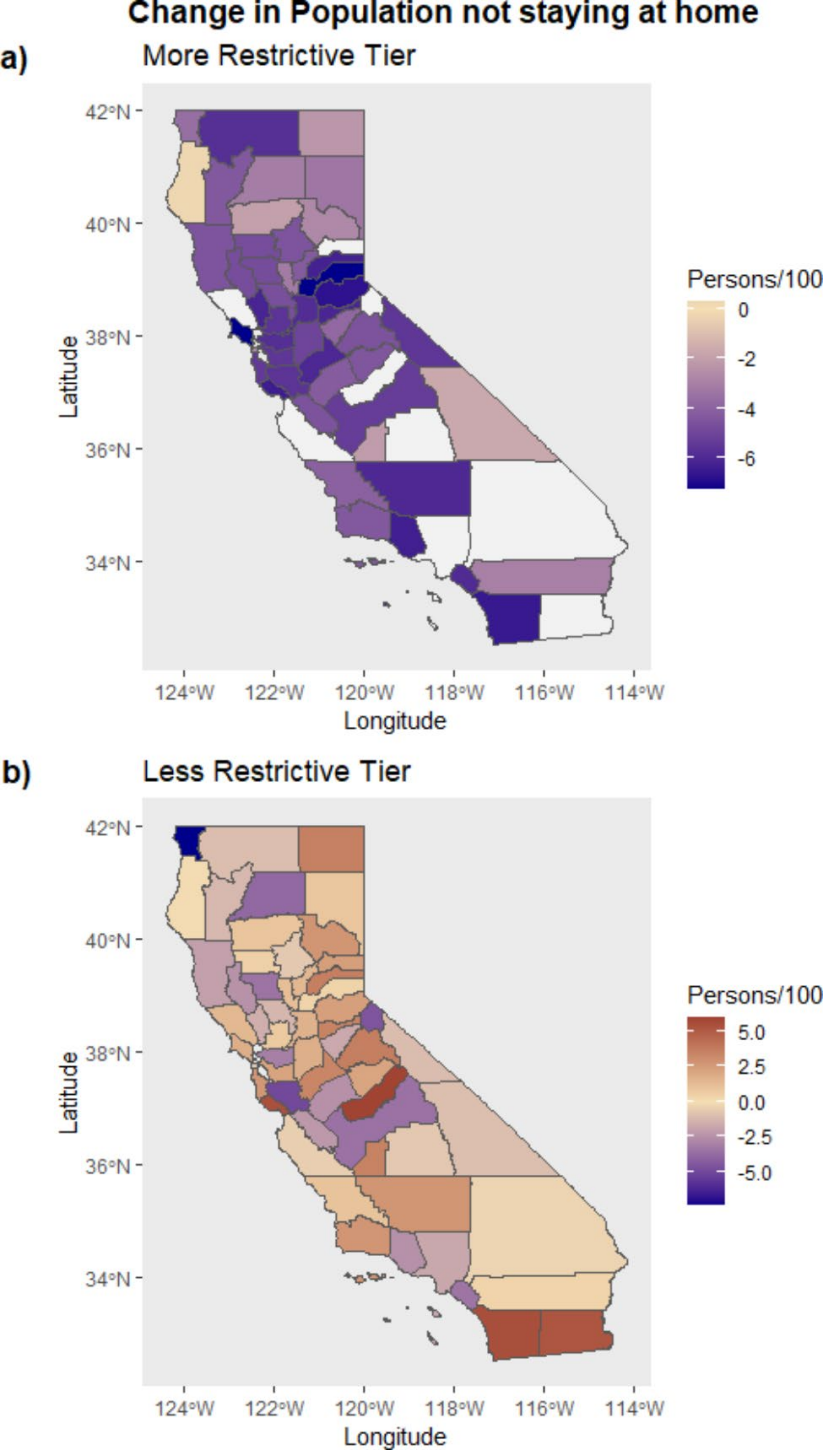
Change in the mobility (population not staying at home per 100 persons) in California counties associated with moving to a more restrictive tier (**a**) or less restrictive tier (**b**). A positive change indicates the population not staying at home per 100 persons (orange), while a negative change indicates the population staying at home per 100 persons (purple)

Generally, moving to a more restrictive tier led to a decrease of -4.45 [-5.44, -3.47] persons not staying at home per 100, while a less restrictive tier increased mobility by 0.57 [-0.08, 1.22] per 100 persons, as intended (Fig. 2). Regions such as Northern California, particularly in the Bay Area and the Eastern Sierras, as well as counties along most of the coastline had a greater decrease in mobility following a change to a more restrictive tier. The counties with the largest decrease in mobility (a change of 6–7 individuals staying at home per 100 persons) after moving to a more restrictive tier include Marin and Placer counties (Fig. 2a). In contrast, the counties that showed the smallest decrease in mobility (a change of 0–3 individuals staying at home per 100 persons) after a change to a more restrictive tier were Del Norte, Modoc, Trinity, Tehama, Plumas, Sutter, Inyo, Kings, and Riverside counties. It is important to note that some counties, such as Los Angeles, remained in the purple tier (most restrictive) for several months and then gradually loosened restrictions but were never reclassified to a more restrictive tier, and therefore could not be analyzed for the increased restrictions change; these counties appear as missing in the map (Fig. 2b).

Differences were observed in trends of increased mobility after moving to a less restrictive tier, although these estimates are less precise. The highest increases in mobility occurred in rural regions such as the northern California coastline and northern mountain ranges (i.e., Shasta Cascades and Sierra Nevada) as well as urban regions including Southern California. In cases where counties went to the least restrictive tier, the counties that most increased their mobility (greater than 5 staying at home per 100 persons) were Imperial, Mono, Sonoma, and Santa Cruz. Counties that had the smallest increase in mobility (0-2.5 staying at home per 100 persons) were Ventura, San Benito, Tulare, Napa, and Del Norte.

### Differences by trips

**Fig. 3:**
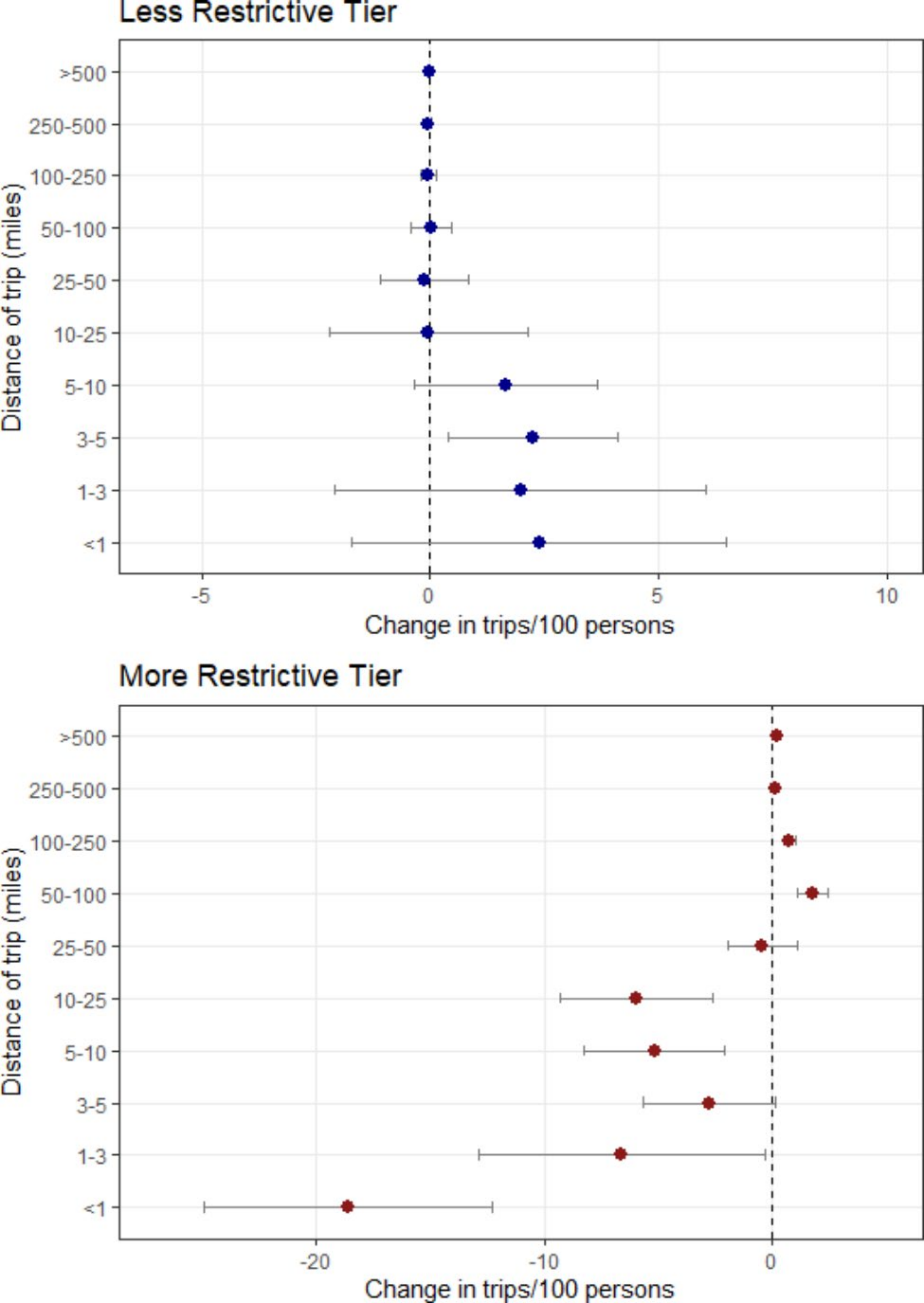
Change in mobility (average daily trips per 100 persons) related to tier system change by the distance of trip in miles across California counties. The points represent the point estimates, and the lines depict the 95% confidence intervals

The changes in mobility varied by the distance of the trips analyzed across all counties as shown in Fig. 3. For changes to a more restrictive tier, the number of trips between 10 and 25 miles per 100 people greatly decreased along with the number of trips between 5 and 10 miles, 3–5 miles, and 1–3 miles. The most significant decrease was in trips less than 1 mile, however, which decreased by an average of 20 daily trips per 100 people. Interestingly, longer trips of 50–100 miles and 100–250 miles were shown to increase following changes to a more restrictive tier. When shifting to a less restrictive tier, there was little change in longer trips of greater than 25 miles per 100 people. In the medium range of trips of 3–5, 5–10, and 10–25 miles in distance, however, there was an increase in each of these categories by approximately 2–3 trips per 100 people. Sensitivity analyses using Wednesdays or Fridays as the first day of the week showed similar results (Table S5 & S6).

### Changes in mobility by demographic characteristics

**Fig. 4:**
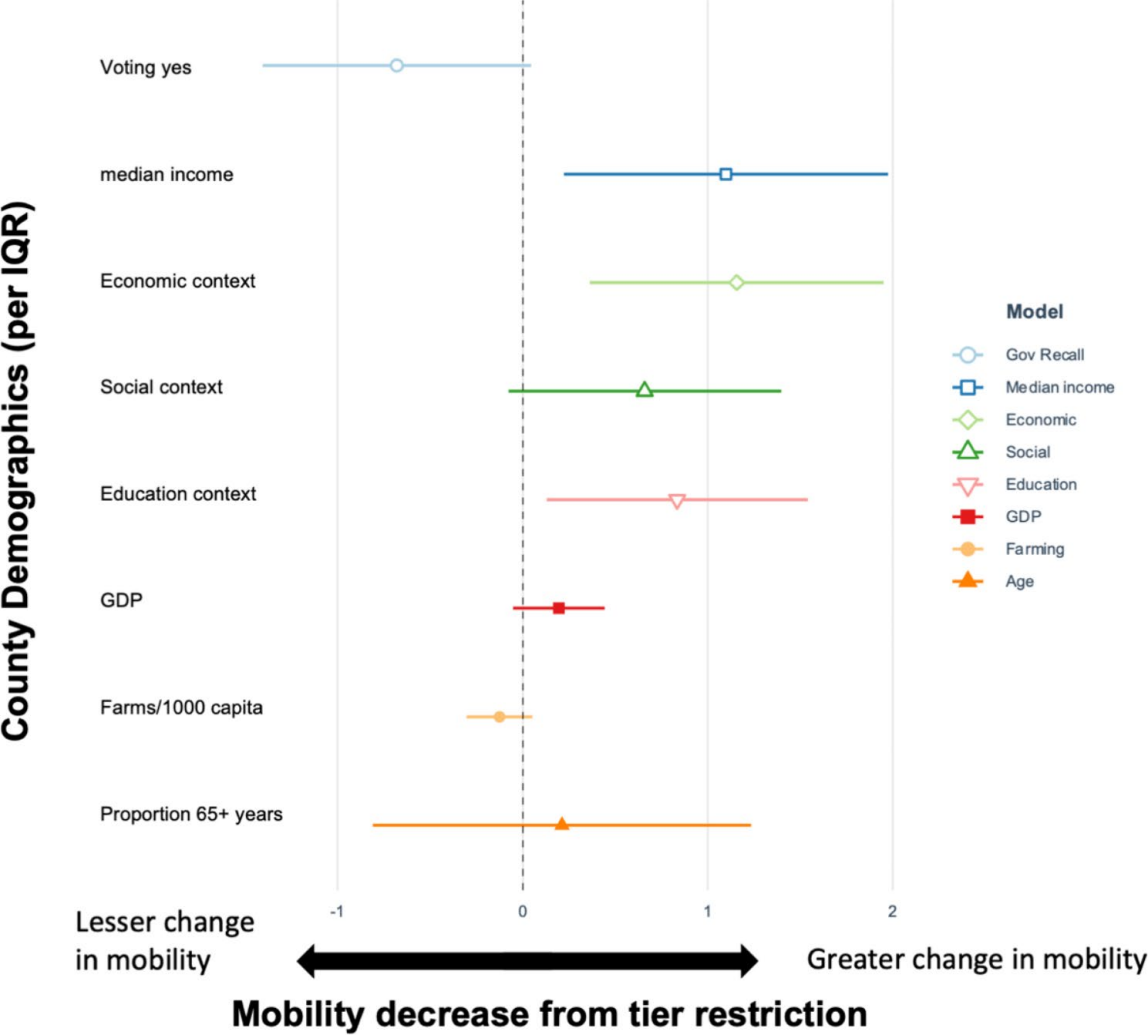
Results of meta-regression showing the association between interquartile range increases in county demographic characteristics (y-axis) and mobility decrease (x-axis) from tier system restrictions in California. Values greater than 0 indicate a greater change in mobility, while values less than 0 indicate a lesser change in mobility

Mobility was affected by tier system changes at the county level with increased restrictions resulting in less population movement. County demographics were associated with the degree of change in mobility observed after moving to a more restrictive tier. For every million USD increase in GDP at the County level, the effect of the tier system on mobility increased by 0.19 [95% CI: -0.06, 0.44] (Fig. 4). Median income had a similar association; counties with a higher income had a greater decrease in mobility when moving to a more restrictive tier. Higher economic, social, and educational context of a county was also associated with a greater change in mobility following tier restrictions. The number of farms per 1000 persons showed a positive association, indicating that when a county has more farms, there was less change in mobility, although this estimate was imprecise (Fig. 4). The proportion of the county that voted for the governor recall also appeared to have a positive association; as the percentage of the county voting yes increased, the change in their mobility patterns was lessened, meaning that counties voting predominantly in favor of the recall were less likely to decrease their mobility when shifting to a more restrictive tier. Lastly, the proportion of persons 65 years and older in a county did not appear to be associated with a greater change in mobility following tier restrictions.

## Discussion

The tier-system implemented by California to restrict mobility during the COVID-19 pandemic was associated with an overall decrease in mobility when counties shifted to a more restrictive tier, and increased mobility when they shifted to a less restrictive tier. We identified that shorter (under 3 miles) and medium range trips (3–50 miles) greatly decreased with changes to a more restrictive tier, whereas only medium trips increased noticeably when moving to a less restrictive tier. Surprisingly, we found that longer trips between 50 and 250 miles increased following a change to a more restrictive tier. Furthermore, important geographic differences across counties regarding mobility patterns were identified, where Northern California and the coastline had a greater decrease in mobility when moving to a more restrictive tier. These differences were in part explained by socio-demographic variations between counties. At the county level, higher GDP, higher education, higher economic context, higher social context, higher income, lower number of farms, and lower percentage of voting yes on the governor recall were associated with a greater impact on mobility. Overall, the tier system proved to be an effective policy in managing mobility, with counties generally changing their mobility patterns following a change in tier status.

We found that the tier system was effective in impacting mobility, reducing the population not staying at home by -4.45 [-5.44, -3.47] per 100 persons when counties moved to a more restrictive tier (Table S5). Similar findings regarding a reduction in mobility with the implementation of restrictive policies were identified in previous studies [7, 22, 23, 26, 29, 32, 35, 37, 38, 40]. For instance, Engle, Stromme, and Zhou (2020) identified a 7.9% decrease in mobility in the United States resulting from the stay-at-home order, Liu and colleagues (2022) observed a decrease in traffic congestion and a 10% lower subway ridership from the lockdown implemented in Chinese cities, Pullano and colleagues (2020) identified a 65% decrease in mobility in France due to the lockdown orders, Wellenius and colleagues (2021) found that in the United States, a state of emergency, social distancing orders, and shelter-in-place orders led to a 9.9%, 24.5%, and 29% decrease in mobility, respectively, and Xiong and colleagues (2020) observed a 5% reduction in mobility from stay-at-home orders in the United States [22, 23, 26, 32, 37]. However, when counties moved to a less restrictive tier, the population not staying at home increased by only 0.57 [-0.08, 1.22] per 100 persons, with imprecise results (Table S6). This indicates that even when restrictions loosen, counties may not revert back to original mobility patterns immediately [54, 55]. Risk perception may play an important role in the willingness of populations to continue to implement protective measures, even with easing of restrictions [56, 57]. Similarly, Borkowski, Jażdżewska-Gutta, and Szmelter-Jarosz (2021) observed that a heightened fear of coronavirus led to larger reductions in travel time and demonstrated that both enforced and self-imposed restrictions led to reductions in daily mobility in Poland [35]. This can also help explain the spatial variation in mobility changes throughout the state, as some counties may have the capacity and resources to maintain more conservative behaviors with regards to COVID-19 (Fig. 3) [46]. This was also highlighted by Chiou and Tucker (2020), where they observed that certain factors contributed to self-isolation behaviors, such as high income and access to high-speed Internet [24]. The results of this study provide insight into the potential impacts of implementing a spatially derived policy, like the tier-system, in efforts to combat dynamic public health emergencies.

The effectiveness of the California tier system policy also varied by distance of the trips traveled. Overall, the daily number of trips decreased by -36.7 [-56.3, -17.0] per 100 persons when moving to a more restrictive tier, with strongest reductions in lower distance trips of 25 miles or less (Table S5). However, results also indicate unintended consequences of this policy as moving to a more restrictive tier increased trips from 50 to 250 miles (Fig. 3). It is possible that this reverse effect may be explained by residents traveling to neighboring counties when moving to a more restrictive tier to avoid restrictions; however, this would have to be further explored with a dataset that includes information on traveling behaviors between counties. This differs from some of the existing research showing that lockdowns have a stronger reduction in long-range mobility than short-range trips [17, 37]. However, previous work on this topic has been limited and varied; Pullano and colleagues identified that lockdown was associated with decreases in shorter trips in France [37], whereas Schlosser and colleagues found that in Germany, long distance trips decreased more strongly than short distance trips following the COVID-19 lockdown [58]. This may be due to differences between the tier system and full lockdown measures evaluated in prior studies. The unintended consequences of the tier system may also be due to limited enforcement. The California tier system was enforced more strictly for businesses, but there was limited enforcement concerning individual adherence to the stay-at-home orders and recommendations to halt travel (Table S2). California did not enforce the law to the same degree other parts of the world did, such as France issuing fines for violations against the home confinement orders and lockdown restrictions [37]. Taken together, this may explain the opposite observed effect of an increase in longer trips. These unintended effects are critical to understanding how the population responded to the policy and to better prepare for future measures to reduce mobility.

The variation in mobility reduction from the tier system between counties in California provides insight into how the policy could be adapted to the demographics of sub-populations to maximize effectiveness. We found that as the county GDP and median income increased, the tier system had a greater impact on mobility. Similarly, as the social, education, and economic context of a county increased based on indicators from the Healthy Places Index [59], mobility had a greater decrease following the change to a more restrictive tier (Fig. 4). Several studies have identified higher compliance with COVID-19 policies among regions with higher income and increased access to resources [24–26, 35, 36, 60–62]. It is important to note that many frontline workers lacked the ability to shelter in place, which in turn increased their risk of contracting, and spreading, the virus [63]. Similar to our results, it has been shown in other studies that areas with lower income had less of a reduction in population mobility during the pandemic [64]. This could potentially be due to the percentages of frontline and essential workers in lower-income communities compared to higher-income jobs that can allow options for remote work. These differences can be partially explained by a mobility adaptation disparity since higher-paying jobs have increased flexibility to work from home when compared to essential positions that dominate the job sector in lower-income counties [60].

Similarly, although the results were not precise, we did find that the number of farms was associated with less of a reduction in mobility when moving to a more restrictive tier (Fig. 2). Farmworkers were shown to be particularly affected by COVID-19 [65]. This may be due to the essential nature of agricultural work; farmworkers were expected to continue working despite tier system restrictions [66]. This population is particularly vulnerable as they tend to have lower incomes, and many are ineligible for unemployment and other benefits. The exploitative work conditions and lack of social protections are important to consider when understanding differing responses to the tier system [66, 67]. Essential workers comprise 26% of the working-age population, and nearly 50% are from minority racial and ethnic groups. Minority groups are also at greater risk of numerous chronic diseases that are also linked to worse COVID-19 outcomes [46]. This is crucial to study as frontline workers and lower-income communities can have higher exposure rates to the virus, driving further health disparities and inequities. COVID-19 measures tailored and adapted to these vulnerable populations are necessary to effectively and equitably implement public health policies and limit viral spread [30, 46, 68].We also found that the county-level recall election results were associated with tier system response. In other words, counties voting for the recall were less likely to have a decrease in mobility when moving to a more restrictive tier (Figure S1). In California, the recall election emerged in part as a result of the opposition to the Governor’s COVID-19 policies, including the tier system. The consideration of politics is critical to understanding the effects of COVID-19 policies, as the social and political economy are key to shaping compliance to public health measures [69].

Results of this study can be used to inform measures and policies to continue to combat the ongoing COVID-19 pandemic. In 2023, California is in a different phase in COVID-19 response than when the tier system was first implemented, but there continues to be a strong need to tailor actions to best adapt to the evolving evidence and understanding regarding viral transmission and epidemic response. Results of this work highlight the importance of not only informing policies and response measures based on local epidemiological information, but also considering the social context and vulnerability of a community at a fine spatial scale. A “one size fits all” approach to mobility restrictions will not be the most effective, and considering the specific resources and capacity to adapt to these policies will be critical. This can be important in informing future policies in this constantly evolving pandemic context and can also be useful in the development of other social and health related policies. Considering the specific vulnerability and adaptive capacity of local populations to restrictive measures and public policies is essential to their acceptability and effectiveness. There are limitations to this study that are important to acknowledge. First, as the mobility dataset started in 2019, we only had one year to draw comparisons. Ideally, we would have had more years to use as a baseline mobility measure, but we feel contrasting 2020 and 2021 to 2019 remains useful, as 2019 was not affected by the pandemic or related policies. Second, the mobility data used is experimental, and data quality standards may be lacking. By comparing mobility within each county using the same data source, we feel the data are sufficiently reliable for the purpose of this study. Also, any residents that do not own or go out with a mobile phone will be excluded from the sample; this could produce a bias as, for example, older persons may be less likely to own or travel with mobile phones; phone behaviors may have also changed because of the pandemic. Moreover, any trip that did not have a pause of 10 min or more away from home would not be captured. We also relied on measures of mobility at the county level, and effects may vary within counties; we hope to explore within county variability using more spatially resolved estimates in future work but are currently limited by county-level estimates. Furthermore, it is possible that other factors could be driving mobility other than the tier system, including but not limited to weather. However, as we studied the effect of tier system changes over a ten-month period across various seasons and we use the specific timing of these changes at the county level, it is unlikely that weather patterns would consistently correspond to the timing of the tier system changes and explain the observed effect. There may be residual confounding from exogenous factors but due to capitalizing on the temporality of the tier system across various geographical regions, we think that this would minimally affect our results. We also did not assess the influence of vaccine coverage on the relationship between the tier system and changes in mobility; however, tier changes incorporated vaccine coverage from April 20th, 2021, and on. Future studies may wish to examine modification by vaccine coverage. Also, future work could consider the potential increased effect of counties skipping tiers on population mobility to evaluate the effectiveness of greater change in restrictions, although an alternative methodological approach may be necessary to explore this in a small sample since few counties skipped tiers. It would also be interesting to explore mobility for those moving between counties as well as those moving across states; this would have to be explored with a dataset which provides this information. Lastly, the effects of the pandemic coincided with wildfires and heat waves that affected the state during the summer and fall of 2020, which could also impact mobility; future work could disentangle the specific effect of these events.

In conclusion, we found strong evidence that the California tier system and associated restrictions were effective in decreasing population mobility. However, results also showed unintended effects of increased travel for longer trips when moving to a more restrictive tier, which is important to consider when developing and implementing future policies. It may indicate that greater coordination is required between neighboring counties. There was also spatial variation in the effectiveness of this policy, which can be partially explained by differences in economic activity and political opinions across the state. It is important to understand this heterogeneity in the response to the California tier system policy to adapt it to maximize equity and effectiveness. To our knowledge, this was the first study assessing the impact of the tier system policy in California on mobility patterns during its complete duration and how these mobility patterns differed by various county-level characteristics. Results provide evidence that the regional tier system classification was effective in limiting population mobility during a pandemic. Evaluating the strengths and weaknesses of COVID-19 response policies is informative for other states and countries to increase preparedness and to inform effective policies for future global health emergencies.

## Electronic supplementary material

Below is the link to the electronic supplementary material.

Supplementary Material 1

## Data Availability

The datasets generated during and/or analyzed during the current study are available in the GitHub repository, https://github.com/benmarhnia-lab/CA_tier_system_mobility.git.
