# Supplementary material for: The role of the California tier system in controlling population mobility during the COVID-19 pandemic
Source: BMC Public Health. 2023 May 18;23:905. doi: 10.1186/s12889-023-15858-7 (PMC10195649; doi:10.1186/s12889-023-15858-7)

**Title:** The role of the California tier system in controlling population mobility during the COVID-19 pandemic

**Supplementary Appendix**

**Table S1:** Literature review of existing studies assessing mobility impacts from COVID-19 policies. Information about the authors, title, location, data collection, and results are provided below.

**Table S2:** California Blueprint for a Safer Economy Activity and Business Tiers (CDPH, 2021)

**Table S3:** Descriptive statistics for each county in California.

**Table S4:** Baseline measures of weekly average mobility and standard deviation (SD) per 100 persons for 2019, 2020 and 2021.

**Table S5:** Effect estimate and confidence intervals for moving to more restrictive tier of main results of and sensitivity analyses considering Wednesday and Friday as first day of the week.

**Table S6:** Effect estimate and confidence intervals for moving to less restrictive tier of main results of and sensitivity analyses considering Wednesday and Friday as first day of the week

**Figure S1:** Scatter plot and line of best fit of association between the percentage of voters for each County that voted Yes in 2021 California governor recall election and change in mobility (population staying at home/100 persons) related to tier system restrictions.

**Table S1:** Literature review of existing studies assessing mobility impacts from COVID-19 policies. Information about the authors, title, location, data collection, and results are provided below.

| **Authors** | **Title** | **Location** | **Data Collection** | **Results** |
| --- | --- | --- | --- | --- |
| Beria, P. & Lunkar, V. | Presence and mobility of the population during the first wave of Covid-19 outbreak and lockdown in Italy | Italy | Facebook Data for Good | Movement of people drastically declined at the start of the pandemic, prior to the implementation of lockdown measures. This was also followed by a decrease in movement during a lockdown. |
| Bonaccorsi, G. et al. | Economic and social consequences of human mobility restrictions under COVID-19 | Italy | “Disease Prevention Maps” provided by Facebook through its “Data for Good” program | Mobility is more influenced by lockdowns in cities that have higher fiscal capacity, and lockdowns have the highest effect in decreasing mobility in places with high income inequality. This paper highlights the importance of accounting for these effects. |
| Borkowski, P., Jazdzewska-Gutte, M., & Szmelter-Jarosz, A. | ​​Lockdowned: Everyday mobility changes in response to COVID-19 | Poland | CATI survey | There were significant decreases in travel during the epidemic regardless of age or gender. This depended on many factors including occupation, fear of coronavirus, and household size. From this study, both enforced and self-imposed mobility restrictions were found to be effective. |
| Chakraborty, M. et al | Analysis and Prediction of Human Mobility in the United States during the Early Stages of the COVID-19 Pandemic using Regularized Linear Models | USA | COVID-19 impact analysis platform developed at UMD’s Maryland Transportation Institute | Authors found that daily trips were influenced significantly when travel restrictions, mask-wearing policies, and stay-at-home orders are in place. |
| Chiou, L. & Tucker, C. | Social Distancing, Internet Access, and Inequality | USA | Data from Safegraph of 20 million mobile devices | High income and high-speed Internet were two main forces that led to people staying at home. Since these are interconnected, this is a potential explanation for inequalities that are visible in self-isolation behavior. |
| Coven, J. & Gupta, A. | Disparities in Mobility Responses to COVID-19 | USA- New York | Smartphone Users | With regards to sheltering-in-place adherence, lower-income, Hispanic and African American neighborhoods had more out-of-tract activity during the day and also at nighttime in comparison to affluent areas. This may be the case because minority and lower-income individuals predominantly occupy frontline occupations. Moreover, increased nighttime activity may be a result of an increase in later shifts or needing to visit retail locations while affluent areas may be opting to utilize delivery options. Thus, minority and lower-income communities are more likely to be exposed to COVID through their mobility pattern. |
| Dainton, C. & Hay, A. | Quantifying the relationship between lockdowns, mobility, and effective reproduction number (Rt) during the Covid-19 pandemic in the Greater Toronto Area | Canada | Google Mobility Data | The first two weeks of lockdown showed to have the most drastic impacts on mobility. After that, mobility increased in the summer and decreased in the winter. The second wave didn’t show as much mobility change as the first. |
| Engle, S., Stromme, J., & Zhou, A. | Staying at Home: Mobility Effects of COVID-19 | USA | Unacast (GPS signals from mobile devices), daily cases/social-distancing policies from New York Times, & Demographic data from the MIT Election Data and Science Lab | A stay-at-home order led to a 7.87% decrease in mobility. Counties that had a high percentage of the population over 65, high population density, and low votes for the republican party were more responsive to mobility restriction measures. |
| Kraemer, M.U.G. et al. | The effect of human mobility and control measures on the COVID-19 epidemic in China | China | Real-time human mobility data from Baidu Inc | Human mobility data early on was a good indicator of spatial distribution of Covid cases in China, but after control measures were implemented growth rates became negative. This study showed that control measures in China helped prevent the spread of Covid. |
| Lasry, A. et al. | Timing of Community Mitigation and Changes in Reported COVID-19 and Community Mobility — Four U.S. Metropolitan Areas, February 26–April 1, 2020 | U.S. (4 cities: Seattle, San Francisco, and New Orleans, and from the five boroughs of New York City) | SafeGraph (percentage of personal mobile devices leaving home) | During February 26–April 1, 2020, as cumulative cases increased and community mitigation policies were implemented, community mobility declined in four U.S. metropolitan areas. With the exception of emergency declarations, which were implemented as cases increased in other regions and internationally, these policies were implemented during the period when case counts were increasing in each location, but the timing in relation to cumulative case counts varied. Public policies to increase compliance with social distancing, including limits on mass gatherings, school closures, business restrictions, and stay-at-home or shelter-in-place orders appear to be associated with decreases in mobility. |
| Liu, Z., et al. | Impacts of the COVID-19 Pandemic on Travel Behavior in Large Cities of China: Investigation on the Lockdown and Reopening Phases | China | Traffic congestion index data and subway ridership data | In a comparison of urban travel pre and during the Covid-19 pandemic, the study found that lockdown reduced traffic congestion and the amount that people rode the subway by 10%. |
| Manica, M. et al. | Impact of tiered restrictions on human activities and the epidemiology of the second wave of COVID-19 in Italy | Italy | Google community mobility reports | The introduction of tiers led to a declining trend in mobility, but it was not as reduced as the initial Covid wave. |
| Marwah, A., Feldman, J., Moineddin, R., Thomas, A. | Population Mobility and Socioeconomic Indicators in California, USA and Ontario, Canada during the Covid-19 Pandemic | USA & Canada | Mobile-device data | Least advantaged populations in both locations showed the highest levels of mobility– which suggests they have less ability to follow stay-at-home orders |
| Mattia, M., et al. | Impact of tiered restrictions on human activities and the epidemiology of the second wave of Covid-19 in Italy | Italy | Google community mobility reports data | As tier restrictions increased, travel outside of the area of residence decreased. With this, Covid-19 transmission also decreased leading to a 36% decrease in hospitalizations. |
| Nikiforiadis, A. et al. | Exploring mobility pattern changes between before, during and after COVID-19 lockdown periods for young adults | Greece | Survey | Covid-19 resulted in a decrease in public transportation use and an increase in walking |
| Nouvellet P. et al. | Reduction in mobility and COVID-19 transmission | Worldwide (52 Countries) | Google and Apple mobility data | Initially, the transmission of Covid decreased with the decrease in mobility. Then, in 80% of the countries they analyzed, when control measures relaxed, it became harder to use mobility as a predictive measure for transmission. When mobility and transmission were correlated before and after measures were put in place, mobility was associated with lower transmission rates after the relaxing of control measures, showing that measures passed to encourage social distancing were effective in showing results. |
| Pullano,G., Valdano, E., Scarpa, N., Rubruchi, S., & Colizza, V. | Population mobility reductions during COVID-19 epidemic in France under lockdown | France | Mobile Phone data through Orange Business | Lockdown orders were effective and led to a 65% decrease in mobility in France, with a particular decrease visible in short-range work trips and long-range recreational trips. Big cities decreased mobility to mainly commuting trips. |
| Santamaria, C. et al. | Measuring the impact of COVID-19 confinement measures on human  mobility using mobile positioning data. A European regional analysis | Europe | Aggregated mobile positioning data | This later  analysis shows that the confinement measures explain up to 90% of the  mobility patterns. |
| Szocska, M. et al | Countrywide population movement monitoring using mobile devices generated (big) data during the COVID-19 crisis | Hungary | Geolocation mobile phone data | Authors found a clear trend with weekdays showing higher levels of mobility, and weekends showing lower levels of mobility. |
| Warren, M.S. & Skillman, S.W. | Mobility Changes in Response to COVID-19 | Worldwide & USA | Mobile device data | Mobility has decreased overall since the start of the pandemic. This is true in the USA and worldwide. |
| Wellenius, G.A. et al. | Impacts of Social Distancing Policies on Mobility and COVID-19 Case Growth in the US | USA | Smartphone users who opted for google location history | State of emergency led to a 9.9% decrease in time spent away from residence, social distancing orders led to 24.5% decrease, shelter in place orders led to 29% decrease. |
| Xiong, C. et al. | Mobile device location data reveal human mobility response to state-level stay-at-home orders during the COVID-19 pandemic in the USA | USA | Cell phone location data | Stay at home orders led to a 5% reduction in mobility. There was a strong spontaneous reduction in mobility before government orders and limited reduction after. |

**Table S2:** California Blueprint for a Safer Economy, Activity and Business Tiers, May 18^th^ 2021 (CDPH, 2021)


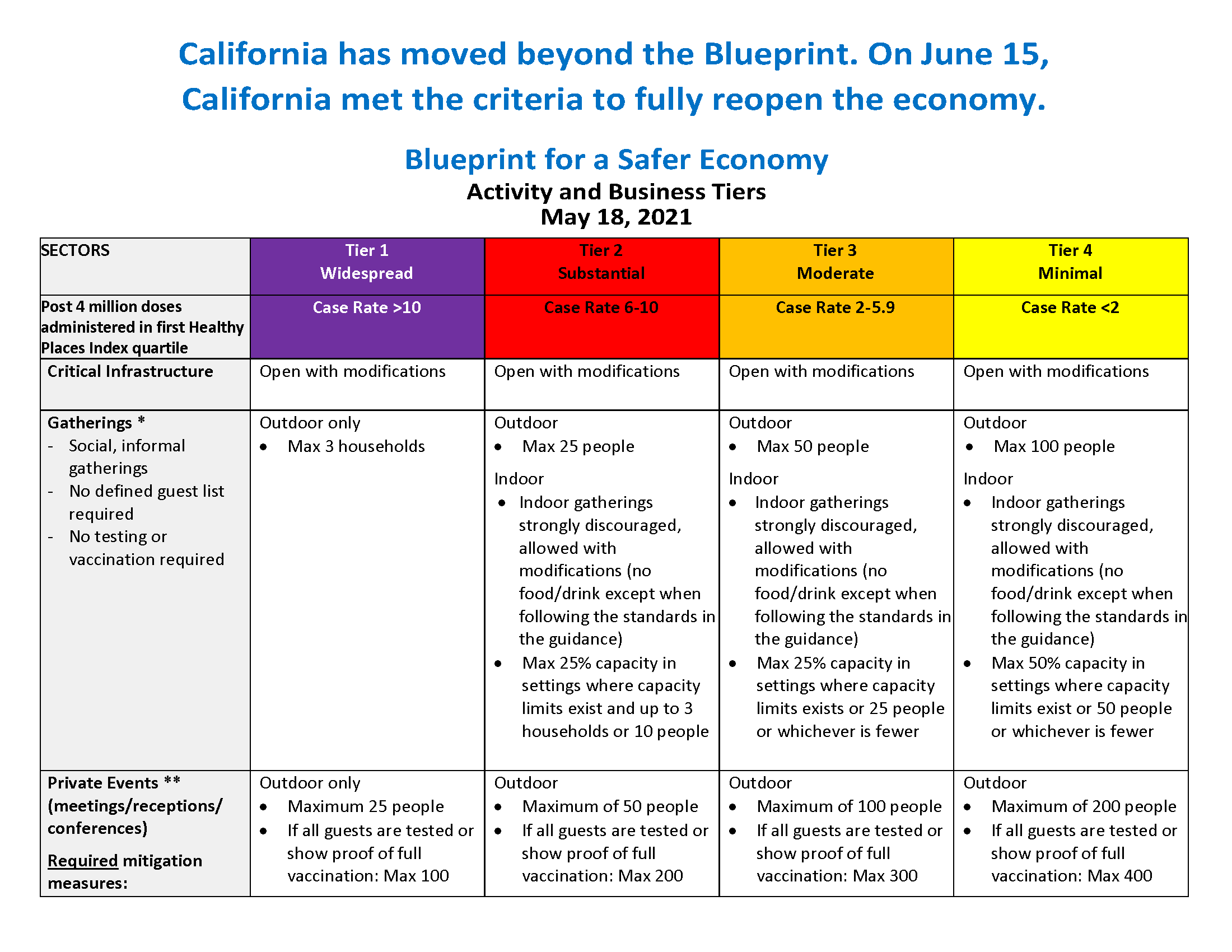


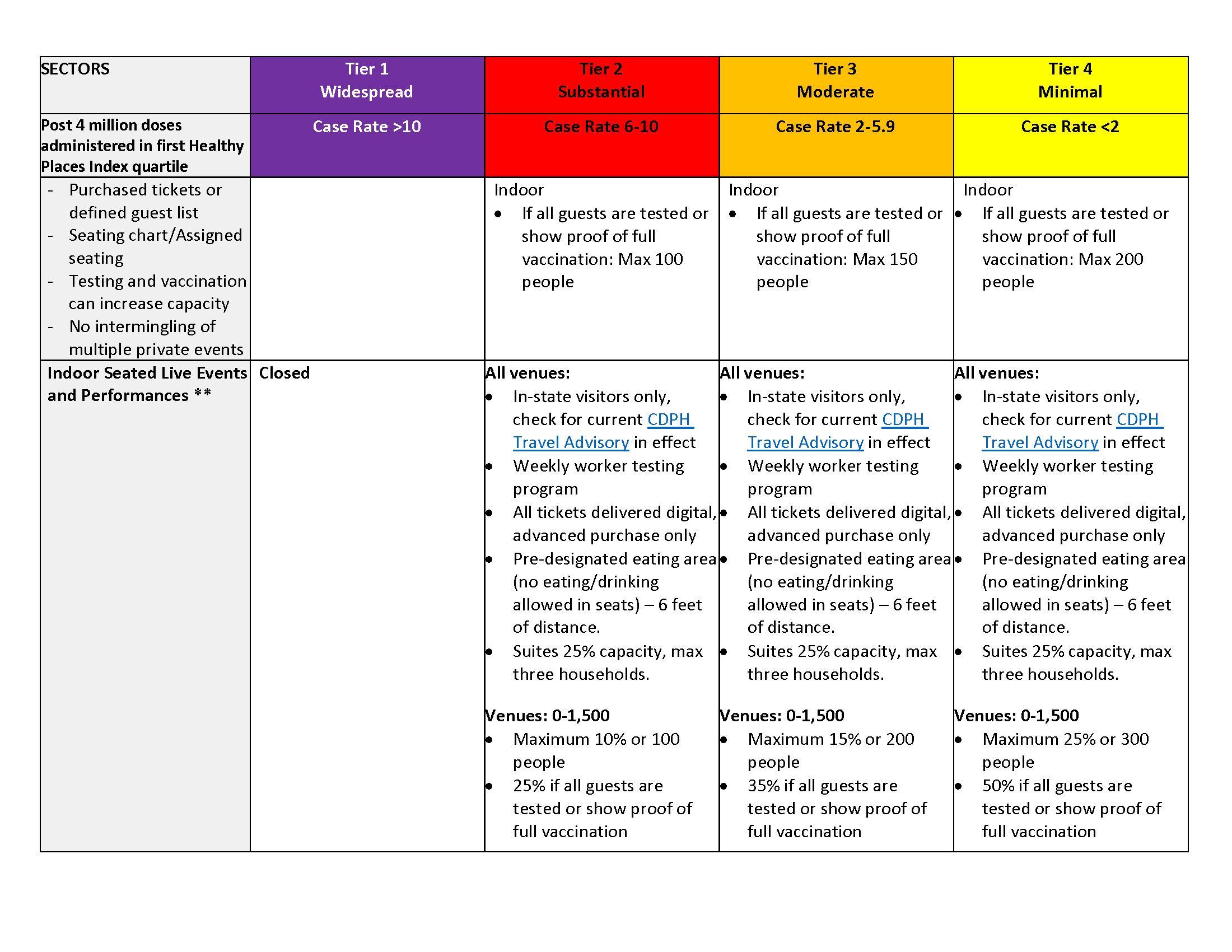


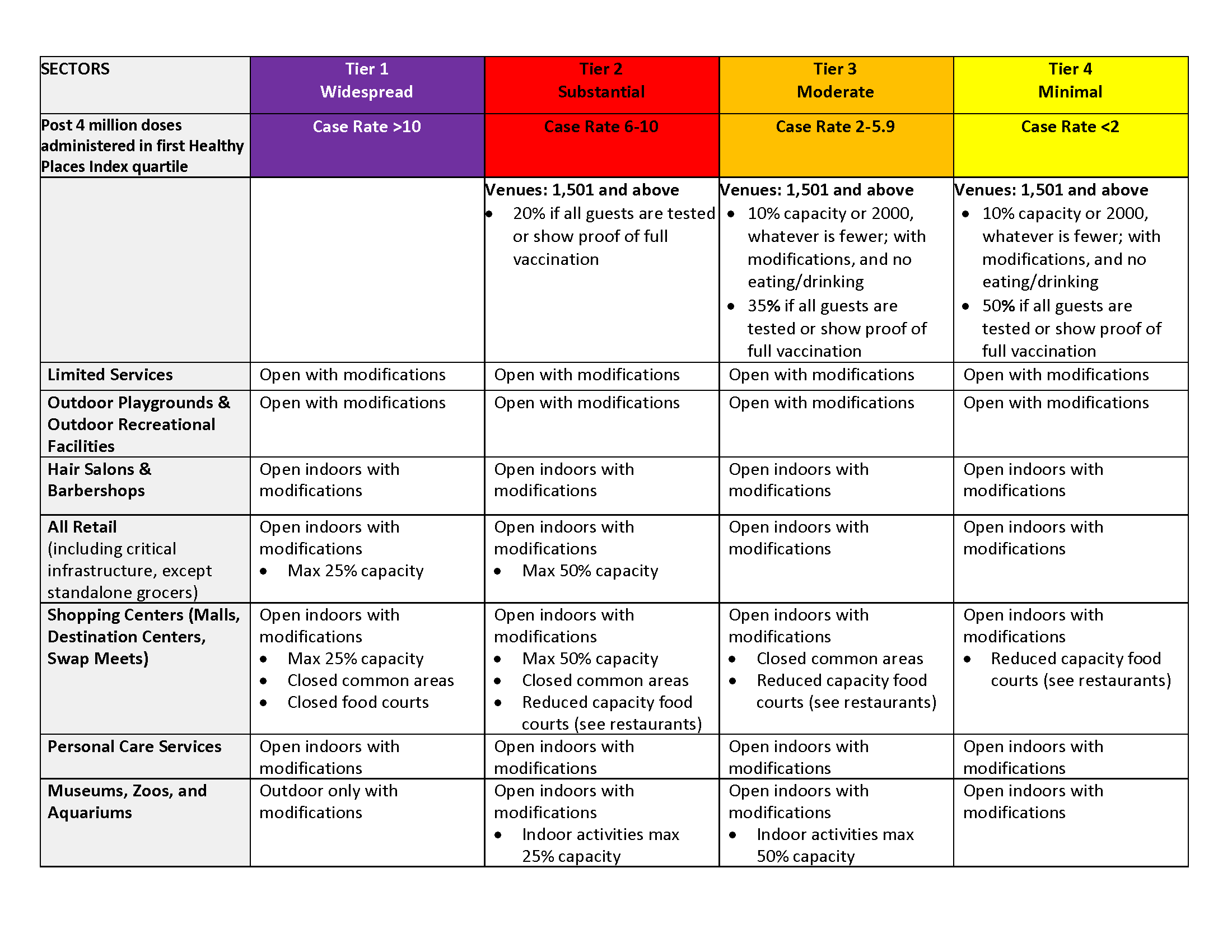


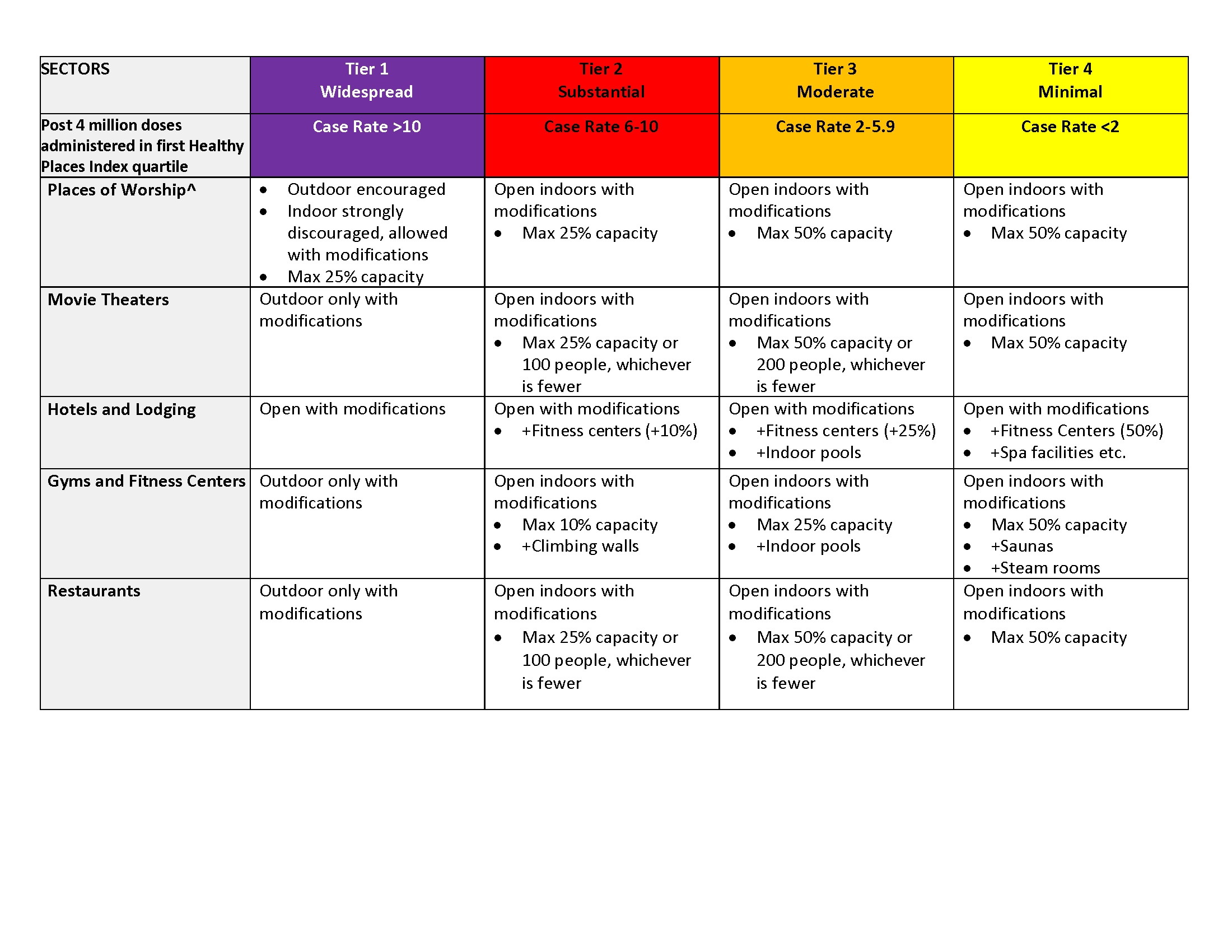


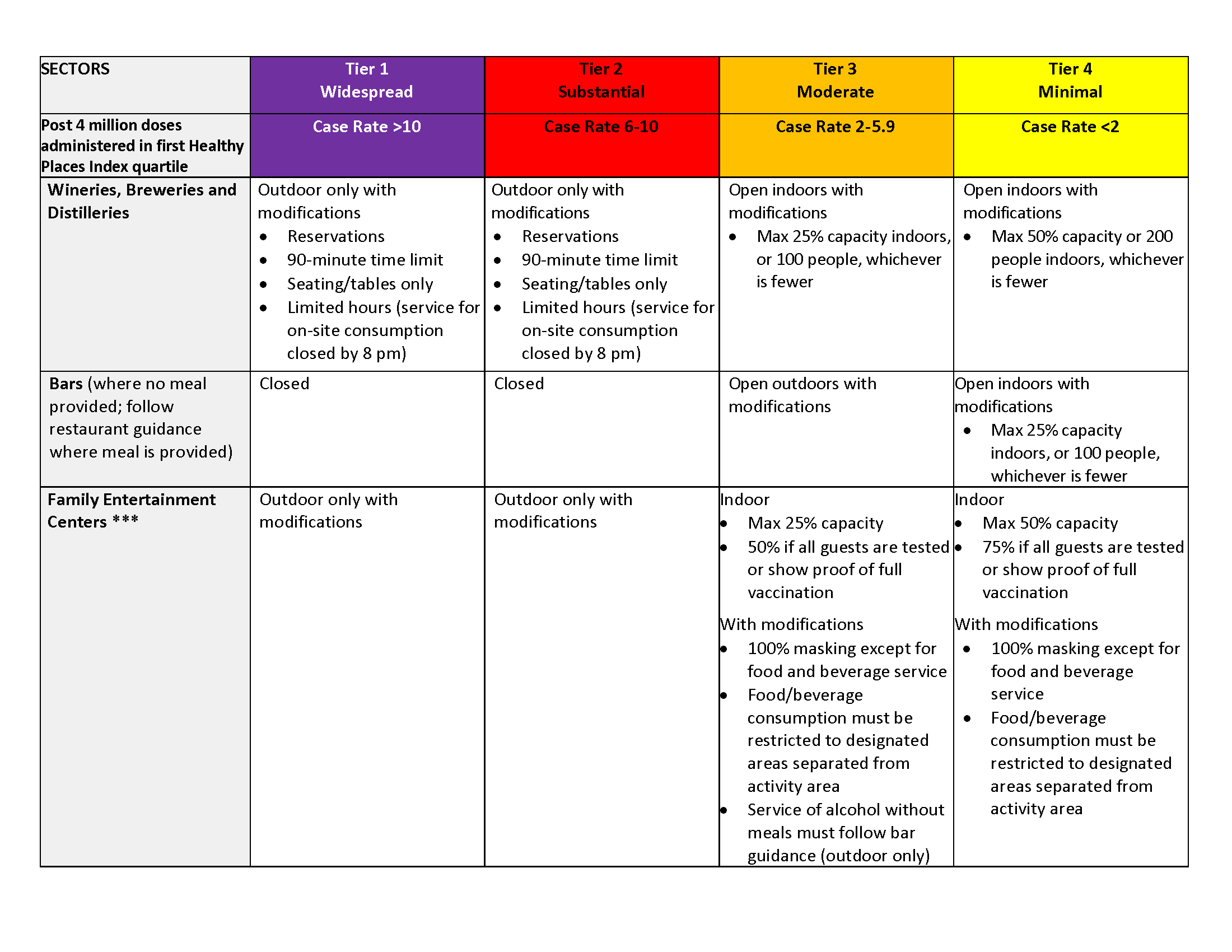


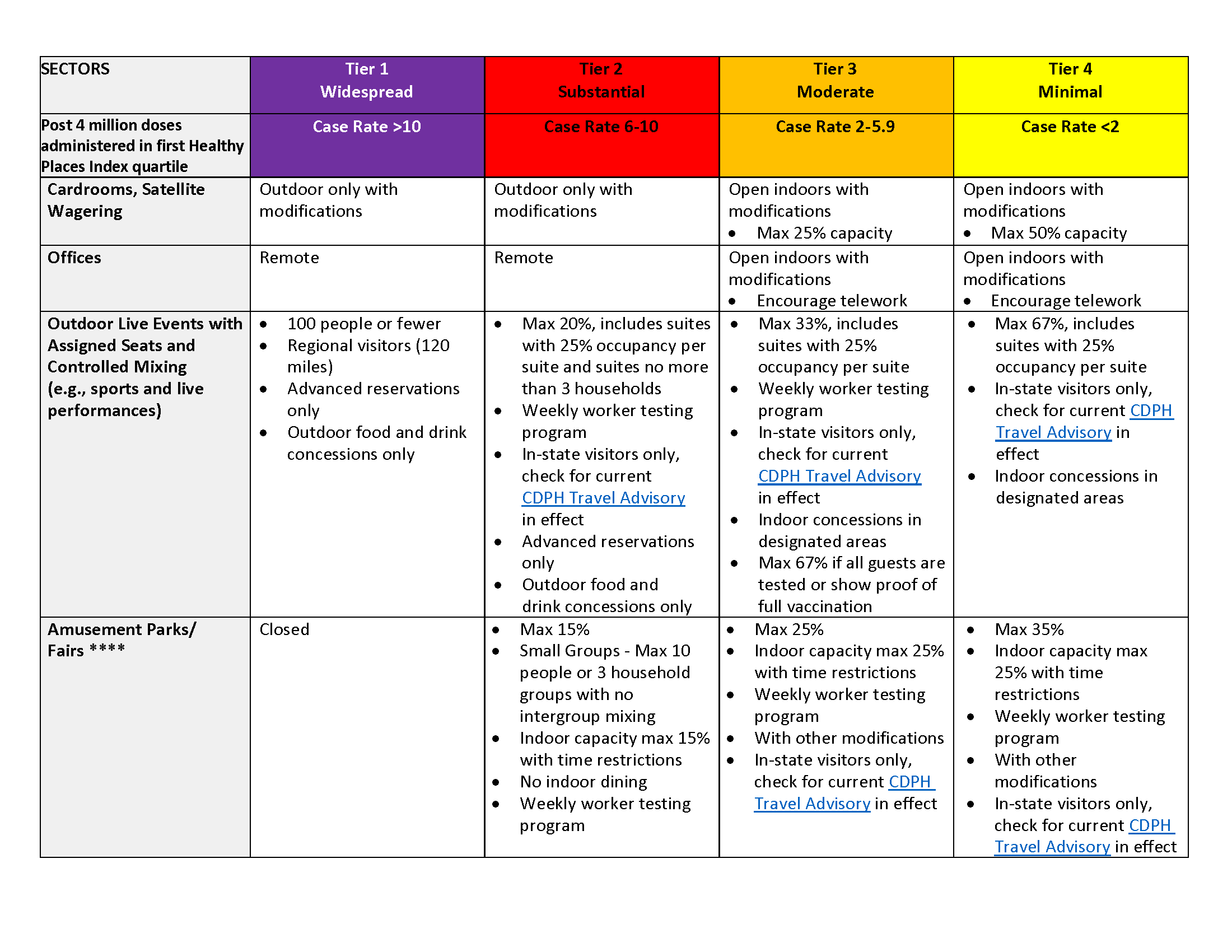


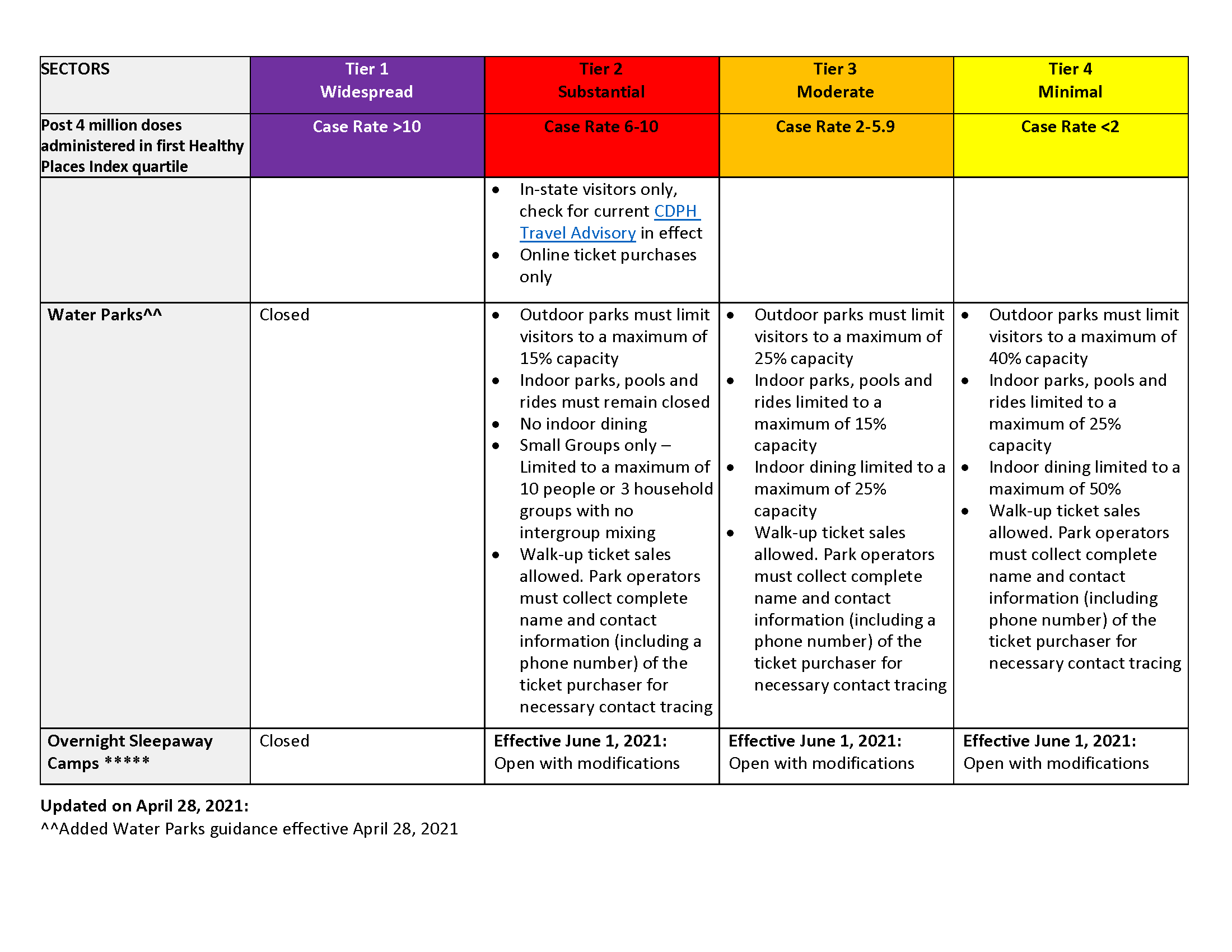


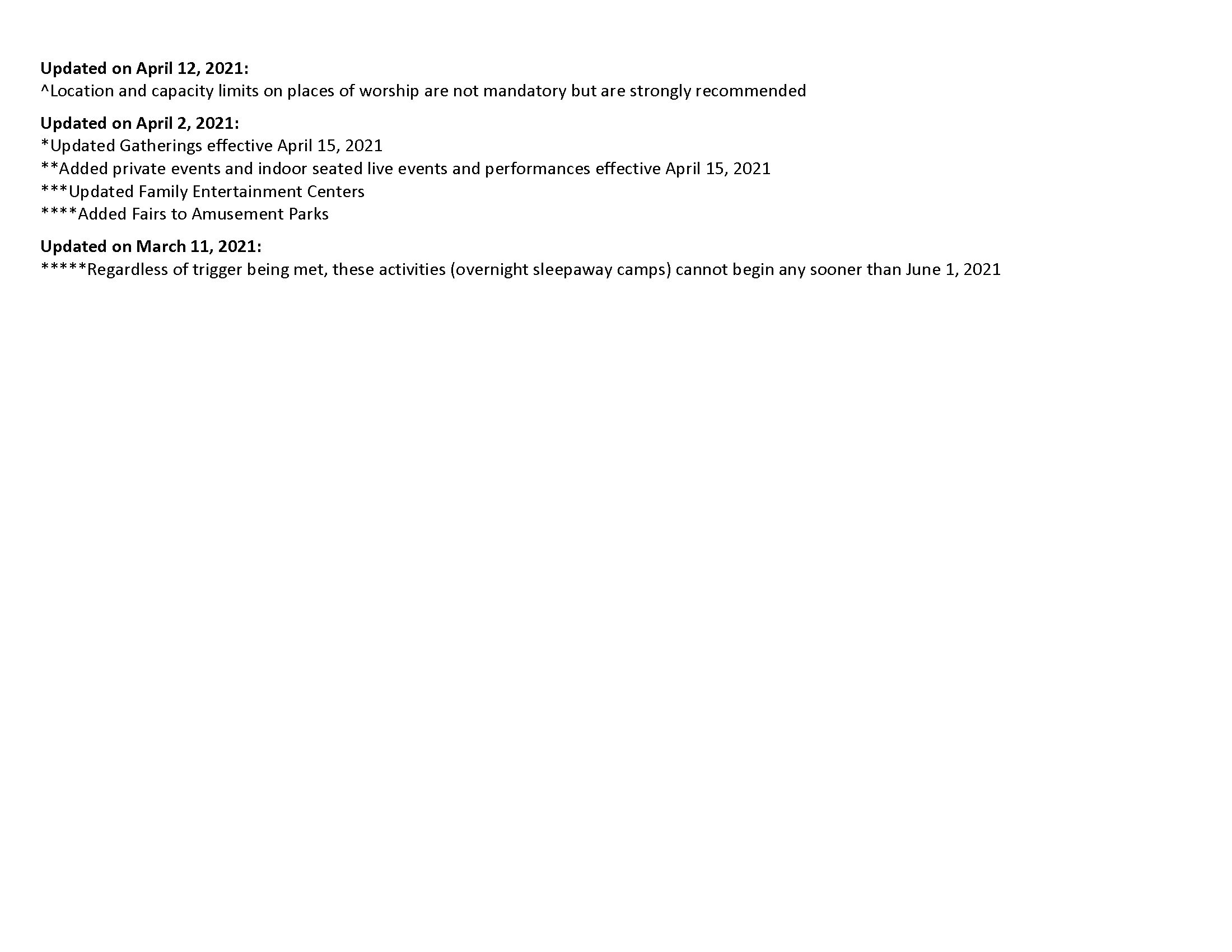


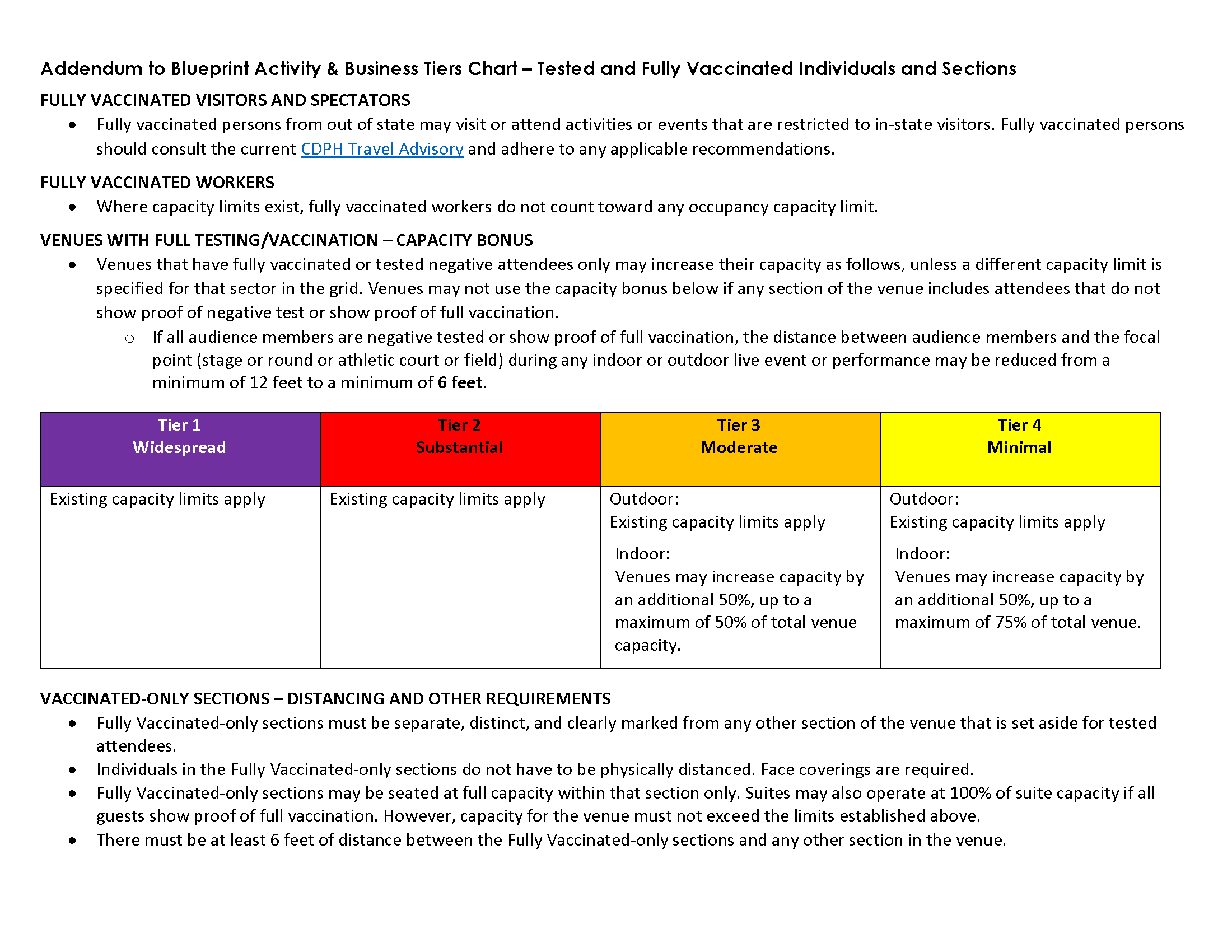


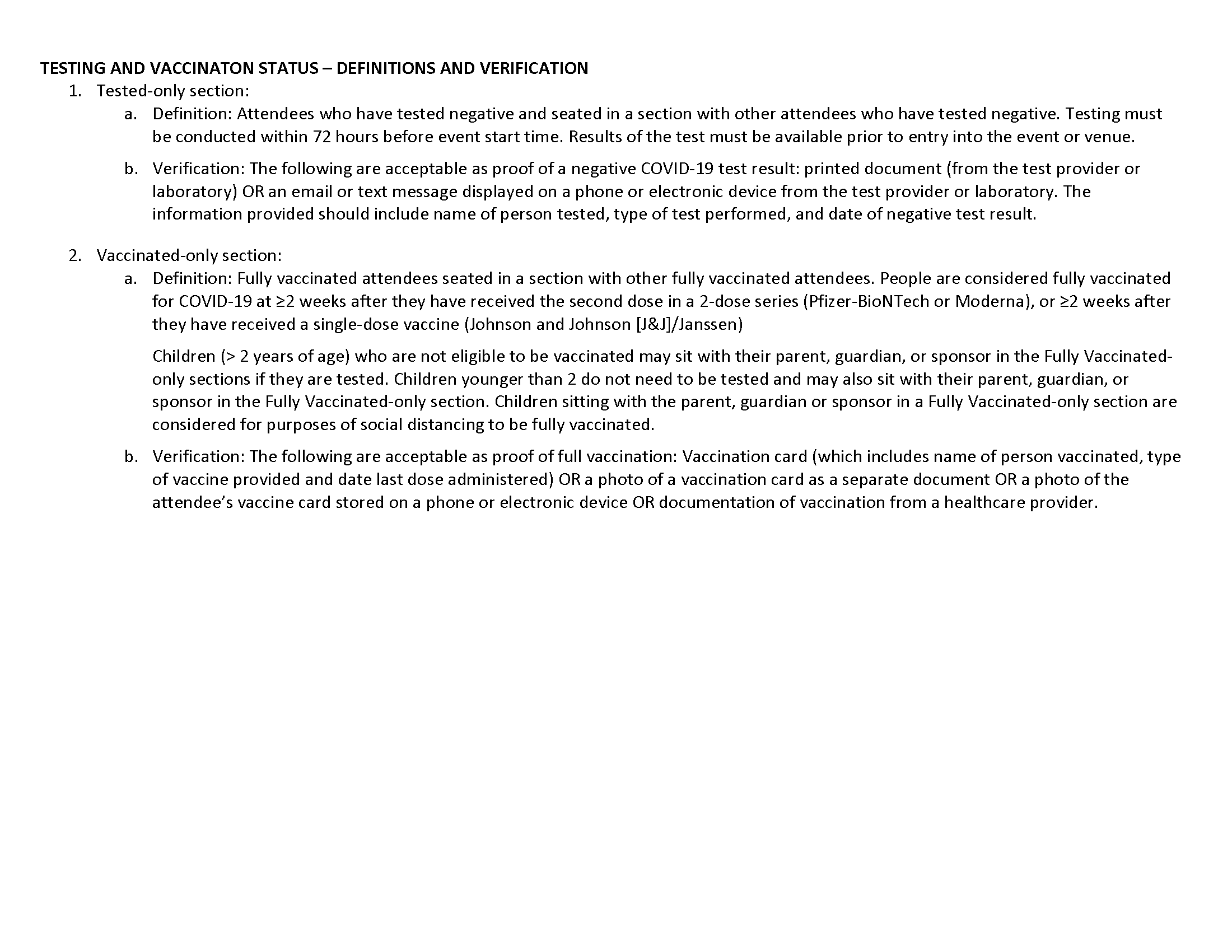


**Table S3:** Descriptive statistics for each county in California.

| **County** | **GDP (billions)** | **Median income (1,000s)** | **Farms (per 100 persons)** | **Pop not staying home (per 100 persons)** | **Percent voted "Yes"** | **Economic** | **Education** | **Social** | **Age 65+** |
| --- | --- | --- | --- | --- | --- | --- | --- | --- | --- |
| Alameda | 144 | 108.32 | 0.162 | 69.85 | 19 | 0.44230806 | 0.27601472 | 0.2138016 | 14.3 |
| Alpine | 0.23 |  | 8.86 | 78.03 | 39 |  |  |  |  |
| Amador | 1.86 |  | 5.99 | 73.3 | 65 | -0.3260527 | -0.1441872 | 0.63522802 |  |
| Butte | 10.3 | 62.56 | 2.59 | 71.41 | 53 | -0.6492911 | -0.1773059 | 0.1239706 | 18.5 |
| Calaveras | 1.64 |  | 4.84 | 77.16 | 64 | -0.4397389 | -0.7338436 | 0.25287022 |  |
| Colusa | 1.72 |  | 25.02 | 73.79 | 63 | -0.3546016 | -0.3905429 | 0.47724111 |  |
| Contra Costa | 82.4 | 107.14 | 0.19 | 70.61 | 28 | 0.50784605 | 0.29970098 | 0.53413039 | 16.200001 |
| Del Norte | 1.05 |  | 0.04 | 75.43 | 58 | -1.1532933 | -0.2975483 | -0.386438 |  |
| El Dorado | 8.77 | 87.1 | 0.33 | 71.49 | 57 | 0.18287378 | 0.07484886 | 0.69598569 | 21.799999 |
| Fresno | 47.4 | 57.52 | 0.96 | 73.16 | 50 | -0.6271223 | -0.4053641 | -0.6104609 | 12.6 |
| Glenn | 1.48 |  | 21.1 | 76.48 | 72 | -0.7542151 | -0.666173 | 0.50915147 |  |
| Humboldt | 6.82 | 51.66 | 2.29 | 70.82 | 34 | -0.6087292 | -0.0698833 | -0.0966507 | 18.5 |
| Imperial | 8.44 | 48.47 | 2.47 | 75.12 | 37 | -0.8994041 | -0.2994973 | -0.516044 | 13.1 |
| Inyo | 1.35 |  | 2.99 | 70.22 | 54 | 0.06415085 | -0.0697818 | 0.36737491 |  |
| Kern | 49.51 | 53.07 | 0.65 | 73.97 | 58 | -0.5637054 | -0.4427024 | -0.308663 | 11.3 |
| Kings | 6.14 | 58.45 | 4.32 | 76.8 | 64 | -0.6717835 | -0.4703317 | -0.4310551 | 10.5 |
| Lake | 2.42 | 47.14 | 0.3 | 72.23 | 40 | -1.0282414 | -0.3756111 | -0.4107785 | 22.299999 |
| Lassen | 1.49 |  | 11.06 | 77.03 | 84 | -0.5529389 | -0.3773835 | 0.24911399 |  |
| Los Angeles | 787.18 | 72.8 | 0.004 | 70.65 | 29 | -0.1103577 | 0.01821149 | -0.3673863 | 14.1 |
| Madera | 7.2 | 64.83 | 1.35 | 76.57 | 60 | -0.7515772 | -0.4607518 | 0.21591228 | 14.5 |
| Marin | 23.65 | 110.84 | 0.46 | 68.74 | 16 | 0.88270845 | 0.68922342 | 1.05324899 | 23 |
| Mariposa | 0.91 |  | 10.46 | 75.44 | 61 | -0.268542 | -0.4539635 | 0.67879989 |  |
| Mendocino | 4.05 | 51.74 | 1.29 | 71.42 | 30 | -0.5415574 | -0.2265638 | 0.07688579 | 23.4 |
| Merced | 9.8 | 61.17 | 4.19 | 75.02 | 50 | -0.8392347 | -0.5308744 | -0.5305694 | 11.4 |
| Modoc | 0.46 |  | 57.46 | 72.38 | 78 | -0.9865037 | -0.8250101 | 0.75026788 |  |
| Mono | 1.14 |  | 6.44 | 70.93 | 43 | 0.03274949 | 0.10122434 | 1.03268009 |  |
| Monterey | 27.42 | 77.51 | 0.48 | 71.97 | 29 | -0.2097414 | -0.3008102 | 0.10562811 | 14 |
| Napa | 11.32 | 92.77 | 0.55 | 69.23 | 30 | 0.46827086 | 0.06282074 | 0.55434788 | 19.6 |
| Nevada | 4.76 | 68.82 | 0.34 | 71.45 | 45 | -0.1380386 | -0.0077039 | 0.799433 | 27.799999 |
| Orange | 260.48 | 95.93 | 0.001 | 70.16 | 48 | 0.3853715 | 0.14221054 | -0.0031491 | 15.3 |
| Placer | 24.75 | 97.72 | 0.29 | 69.56 | 51 | 0.40432248 | 0.11414293 | 0.76896246 | 19.9 |
| Plumas | 1.21 |  | 3.35 | 76.12 | 63 | -0.5679057 | -0.6453061 | 0.38728578 |  |
| Riverside | 90.77 | 73.26 | 0.13 | 73.1 | 49 | -0.2572264 | -0.3766463 | -0.1801364 | 14.8 |
| Sacramento | 96.63 | 72.02 | 0.35 | 72.63 | 37 | -0.159648 | -0.1341707 | 0.0599542 | 14.4 |
| San Benito | 2.76 |  | 2.42 | 75.22 | 42 | 0.25322204 | -0.2424069 | 0.26227083 |  |
| San Bernardino | 96.34 | 67.9 | 0.01 | 74.23 | 50 | -0.4234693 | -0.3739402 | -0.2248766 | 12 |
| San Diego | 245.14 | 83.99 | 0.002 | 71.24 | 42 | 0.05698306 | 0.04880965 | 0.26818697 | 14.5 |
| San Francisco | 183.17 | 123.86 | 0 | 62.24 | 14 | 0.58496391 | 0.58333334 | 0.15537209 | 16 |
| San Joaquin | 32.33 | 69 | 1.05 | 73.67 | 44 | -0.3951485 | -0.3534653 | -0.033272 | 13 |
| San Luis Obispo | 18.52 | 77.27 | 2.51 | 72.24 | 40 | 0.1151324 | 0.12653774 | 0.63741929 | 21.1 |
| San Mateo | 114.86 | 138.5 | 0.01 | 66.81 | 21 | 0.90602446 | 0.43421083 | 0.65113761 | 16.5 |
| Santa Barbara | 30.19 | 75.65 | 0.51 | 71.93 | 37 | 0.10735793 | -0.0242838 | 0.48368193 | 15.8 |
| Santa Clara | 328.26 | 133.08 | 0.04 | 69.99 | 25 | 0.75349984 | 0.37522616 | 0.68434359 | 13.8 |
| Santa Cruz | 15.31 | 89.27 | 0.08 | 70.7 | 21 | 0.19608736 | 0.05185719 | 0.31378468 | 17.6 |
| Shasta | 8.72 | 63.09 | 0.78 | 73.38 | 67 | -0.5682257 | -0.2522869 | 0.32998984 | 21.1 |
| Sierra | 0.23 |  | 17.3 | 81.67 | 63 | -0.7379593 | -4.727192 | 0.55460505 |  |
| Siskiyou | 1.94 |  | 13.67 | 75.14 | 62 | -0.8963563 | -0.3025763 | 0.3235485 |  |
| Solano | 26.44 | 86.65 | 1.13 | 73.03 | 37 | 0.10179284 | -0.1837341 | -0.0456081 | 16.200001 |
| Sonoma | 31.96 | 87.83 | 0.45 | 70.81 | 23 | 0.24739207 | 0.04956621 | 0.71693327 | 20.700001 |
| Stanislaus | 25.13 | 63.04 | 1.61 | 73.2 | 53 | -0.5088273 | -0.467733 | -0.1725471 | 13.4 |
| Sutter | 3.83 | 62.78 | 5.19 | 76.68 | 59 | -0.4378567 | -0.3219018 | 0.31801855 | 15.6 |
| Tehama | 2.42 | 53.48 | 3.7 | 75.68 | 69 | -0.7989501 | -0.2177713 | 0.31389595 | 20.4 |
| Trinity | 0.57 |  | 0.57 | 73.32 | 54 | -0.9847755 | -0.2268227 | 0.13877163 |  |
| Tulare | 19.14 | 57.69 | 2.81 | 74.02 | 60 | -0.7622604 | -0.4417159 | -0.1351815 | 11.5 |
| Tuolumne | 2.77 |  | 3.16 | 72.4 | 62 | -0.4655659 | -0.0658995 | 0.31823354 |  |
| Ventura | 59.63 | 92.24 | 0.13 | 71.13 | 41 | 0.4142528 | 0.02134083 | 0.41395504 | 16.1 |
| Yolo | 15.34 | 71.42 | 2.89 | 72.84 | 29 | -0.1535246 | 0.2145393 | -0.0065253 | 12.9 |
| Yuba | 3.39 | 58.77 | 2.76 | 75.58 | 61 | -0.8740416 | -0.2214422 | -0.2794641 | 13.1 |

**Table S4:** Baseline measures of weekly average mobility and standard deviation (SD) per 100 persons for 2019, 2020 and 2021.

| Outcome | 2019  Mean (SD) | 2020  Mean (SD) | 2021 (Jan-Jul)  Mean (SD) |
| --- | --- | --- | --- |
| Population not staying at home (per 100) | 79.61 (2.59) | 73.37 (4.89) | 74.72 (5.52) |
| Number of trips  (per 100 persons) | 432.38 (68.12) | 259.23 (60.26) | 279.42 (46.0) |
| <1 mile | 107.57 (29.95) | 63.78 (23.63) | 72.02 (19.30) |
| 1-3 miles | 119.63 (32.71) | 65.97 (18.29) | 68.39 (14.77) |
| 3-5 miles | 49.31 (14.31) | 28.27 (8.15) | 31.30 (7.43) |
| 5-10 miles | 54.20 (18.59) | 31.97 (9.08) | 36.73 (10.53) |
| 10-25 miles | 61.47 (19.80) | 37.75 (13.31) | 40.98 (13.12) |
| 25-50 miles | 26.80 (11.87) | 19.12 (9.27) | 18.64 (8.26) |
| 50-100 miles | 9.59 (5.64) | 8.35 (4.43) | 7.57 (4.76) |
| 100-250 miles | 2.68 (1.60) | 3.18 (1.86) | 2.93 (1.85) |
| 250-500 miles | 0.51 (0.38) | 0.59 (0.44) | 0.54 (0.40) |
| >500 miles | 0.63 (0.53) | 0.25 (0.27) | 0.32 (0.60) |

**Table S5:** Effect estimate and confidence intervals for moving to more restrictive tier of main results of and sensitivity analyses considering Wednesday and Friday as first day of the week.

| Outcome | Main analysis | Sensitivity analysis 1  (week starting Wed) | Sensitivity analysis 2  (week starting Fri) |
| --- | --- | --- | --- |
| Population not staying at home (per 100) | -4.45 [-5.44, -3.47] | -4.05 [-5.03, -3.07] | -4.49 [-5.46, -3.51] |
| Number of trips  (per 100 persons) | -36.67 [-56.28, -17.06] | -28.35 [-47.13, -9.58] | -36.86 [-56.21, -17.51] |
| <1 mile | -19.57 [-25.92, -13.22] | -15.96 [-22.05, -9.87] | -20.15 [-26.46, -13.85] |
| 1-3 miles | -7.32 [-13.54, -1.10] | -5.41 [-11.4, 0.59] | -7.25 [-13.41, -1.1] |
| 3-5 miles | -2.37 [-5.23, 0.49] | -1.71 [-4.49, 1.07] | -2.27 [-5.14, 0.6] |
| 5-10 miles | -4.66 [-7.74, -1.57] | -3.55 [-6.56, -0.53] | -4.32[-7.38, -1.26] |
| 10-25 miles | -5.93 [-9.28, -2.58] | -4.44 [-7.67, -1.22] | -5.24 [-8.67, -1.82] |
| 25-50 miles | -0.26 [-1.78, 1.25] | 0.42 [-1.07, 1.9] | -0.24 [-1.74, 1.26] |
| 50-100 miles | 1.87 [1.19, 2.54] | 1.77 [1.11, 2.43] | 1.72 [1.05, 2.39] |
| 100-250 miles | 0.76 [0.50, 1.02] | 0.36 [0.1, 0.61] | 0.57 [0.32, 0.83] |
| 250-500 miles | 0.12 [0.04, 0.21] | 0.05 [-0.04, 0.14] | 0.12 [0.03, 0.21] |
| >500 miles | 0.19 [0.06, 0.33] | 0.12 [-0.01, 0.24] | 0.2 [0.07, 0.33] |

**Table S6:** Effect estimate and confidence intervals for moving to less restrictive tier of main results of and sensitivity analyses considering Wednesday and Friday as first day of the week.

| Outcome | Main analysis | Sensitivity analysis 1  (week starting Wed) | Sensitivity analysis 2  (week starting Fri) |
| --- | --- | --- | --- |
| Population not staying at home (per 100) | 0.57 [-0.08, 1.22] | 0.69 [0.05, 1.33] | -0.24 [-0.89, 0.39] |
| Number of trips  (per 100 persons) | 9.42 [-3.34, 22.18] | 5.16 [-7.13, 17.46] | 9.18 [-3.41, 21.78] |
| <1 mile | 2.87 [-1.23, 6.97] | 0.49 [-0.395, 4.04] | 1.08 [-3.04, 5.2] |
| 1-3 miles | 1.65 [-2.42, 5.71] | 1.3 [-2.62, 5.23] | 1.1 [-2.9, 5.1] |
| 3-5 miles | 1.86 [-0.001, 3.73] | 0.74 [-1.08, 2.56] | 1.35 [-0.52, 3.22] |
| 5-10 miles | 3.10 [1.10, 5.11] | 1.48 [-0.49, 3.45] | 2.53 [0.54, 4.51] |
| 10-25 miles | 1.90 [-0.29, 4.08] | 1.19 [-0.92, 3.3] | 2.82 [0.59, 5.05] |
| 25-50 miles | -0.19 [-1.18, 0.81] | 0.12 [-0.85, 1.09] | 0.34 [-0.63, 1.31] |
| 50-100 miles | 0.09 [-0.35, 0.53] | 0.36 [-0.07, 0.8] | -0.008 [-0.44, 0.43] |
| 100-250 miles | -0.07 [-0.24, 0.10] | -0.05 [-0.22, 0.11] | -0.06 [-0.22, 0.11] |
| 250-500 miles | -0.09 [-0.14, -0.03] | -0.03 [-0.09, 0.02] | -0.05 [ -0.11, 0.17] |
| >500 miles | 0.03 [-0.05, 0.11] | -0.02 [-0.09, 0.08] | 0.08 [-0.0003, 0.17] |

**Figure S1:** Scatter plot and line of best fit of association between the percentage of voters for each County that voted Yes in 2021 California governor recall election and change in mobility (population staying at home/100 persons) related to tier system restrictions.


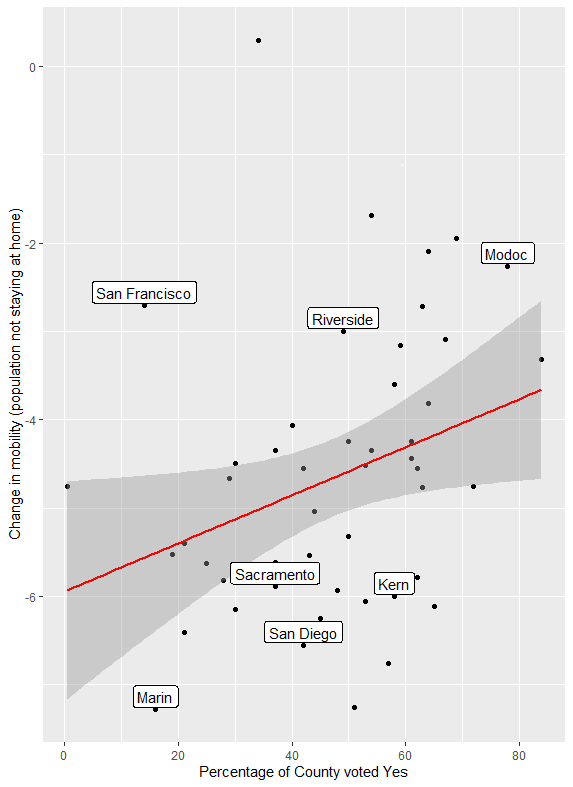

Supplement: Supplementary file 1 — Supplementary Material 1 [file 12889_2023_15858_MOESM1_ESM.docx]
